# Synthesis, Pharmacological Evaluation, and Molecular Modeling of Lappaconitine–1,5-Benzodiazepine Hybrids

**DOI:** 10.3390/molecules28104234

**Published:** 2023-05-22

**Authors:** Kirill P. Cheremnykh, Arkadiy O. Bryzgalov, Dmitry S. Baev, Sergey A. Borisov, Yulia S. Sotnikova, Victor A. Savelyev, Tatyana G. Tolstikova, Shamansur S. Sagdullaev, Elvira E. Shults

**Affiliations:** 1 N.N. Vorozhtsov Novosibirsk Institute of Organic Chemistry, Siberian Branch, Russian Academy of Sciences, Lavrentjev Avenue 9, 630090 Novosibirsk, Russia; cherem@nioch.nsc.ru (K.P.C.); arkadiy@nioch.nsc.ru (A.O.B.); mitja2001@gmail.com (D.S.B.); sergalborisov@mail.ru (S.A.B.); sotnikova@nioch.nsc.ru (Y.S.S.); vicsav@nioch.nsc.ru (V.A.S.); tg_tolstikova@mail.ru (T.G.T.); 2 S.Yu. Yunusov Institute of the Chemistry of Plant Substances, Academy of Sciences of the Republic of Uzbekistan, Mirzo Ulugbek Str. 77, Tashkent 100170, Uzbekistan; plant_inst@icps.org.uz

**Keywords:** diterpenoid alkaloids, lappaconitine, alkynyl ketones, Sonogashira coupling, cyclocondensation, 1,5-benzodiazepines, analgesic activity, antiarrhythmic activity

## Abstract

Diterpenoid alkaloids, originating from the amination of natural tetracyclic diterpenes, have long interested scientists due to their medicinal uses and infamous toxicity which has limited the clinical application of the native compound. Alkaloid lappaconitine extracted from various *Aconitum* and *Delphinium* species has displayed extensive bioactivities and active ongoing research to reduce its adverse effects. A convenient route to construct hybrid molecules containing diterpenoid alkaloid lappaconitine and 3*H*-1,5-benzodiazepine fragments was proposed. The key stage involved the formation of 5′-alkynone-lappaconitines in situ by acyl Sonogashira coupling of 5′-ethynyllappaconitine, followed by cyclocondensation with *o*-phenylenediamine. New hybrid compounds showed low toxicity and outstanding analgesic activity in experimental pain models, which depended on the nature of the substituent in the benzodiazepine nucleus. An analogous dependence was also shown for the antiarrhythmic activity in the epinephrine arrhythmia test in vivo. Studies on the isolated atrium have shown that the mechanism of action of the new compounds is included the blockade of beta-adrenergic receptors and potassium channels. Molecular docking analysis was conducted to determine the binding potential of target molecules with the voltage-gated sodium channel NaV1.5. All obtained results provide a basis for future rational modifications of lappaconitine, reducing side effects, while retaining its therapeutic effects.

## 1. Introduction

**Figure 1:**
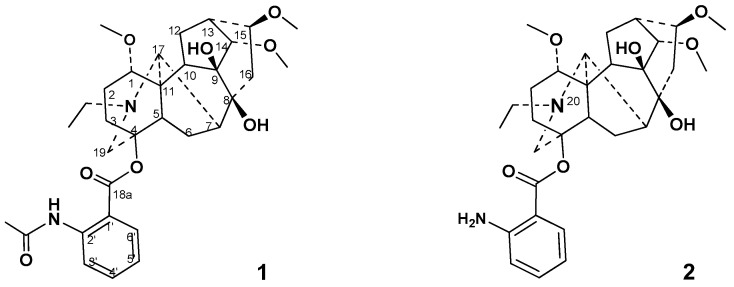
Structure of diterpenoid alkaloids lappaconitine **1** and *N*-deacetyllappaconitine **2**.

Diterpenoid alkaloid lappaconitine **1** (Figure 1) is an important natural drug extracted from the roots of various *Aconitum* and *Delphinium* species [1,2]. It has been used clinically for more than 30 years to treat mild or moderate acute and chronic pains, including cancer-related pain, postoperative pain, and neuropathic pain [3]. Recent studies reported that its analgesic activity is related to a decrease in expression and sensitization of the P2X3 receptors in mice dorsal root ganglion (DRG) neurons [4]. Multiple articles in the medical literature have discussed lappaconitine’s analgesic, anti-inflammatory, arrhythmic/antiarrhythmic, and neurogenic effects [5,6,7,8,9,10]. Regarding the mechanism of action, lappaconitine **1**, its major metabolite *N*-deacetyllappaconitine **2**, and some other alkaloids of *Aconitum* sp. were found to act as sodium channel blockers [11,12,13,14,15,16,17,18]. It was shown that voltage-dependent Na^+^ channel blockers possesses antinociceptive activity and neuronal activity [12,15,18]. In recent years, a large number of studies have investigated the toxicological and pharmacological characteristics of alkaloid lappaconitine **1** and other diterpenoid alkaloids; current reviews [19,20,21,22,23,24,25] have summarized the obtained results.

It was reported that lappaconitine **1** possess certain toxicities [20,23,26] and its oral 50% lethal doses (LD_50_) in mice and rats are 32.4 and 20 mg/kg, respectively [26].

The availability of lappaconitine **1** and *N*-deacetyllappaconitine **2** [24,27] have determined and caused interest in synthesizing new derivatives with reduced toxicity and enhanced pharmacological properties. Studies of the local anesthetic activity and toxicity of aconitum alkaloids and their semi-synthetic derivatives revealed that the presence of an aromatic ring in the C-4 position decreased the toxicity of the alkaloids [28]. A significant variety of lappaconitine derivatives has been synthesized and studied. Most of the pharmacological activity studies have focused on the analgesic, anti-inflammatory, antitumor, and antiarrhythmic effects. Lappaconitine sulfate [29], lappaconitine hydrobromide, and lappaconitine trifluoroacetate [30] have exhibited greater solubility in water and pronounced analgesic activity. Salt formation with sulfonic acid was important for antitumor activity [31,32]. A fatty acid chain at the 8-hydroxy group and 13-OH substituent on the diterpenoid core were favorable for the enhancement of the antiproliferative activity [33]. *N*-Ethyl substituted tertiary amine (N-20), and the saturation state of the ring D of diterpenoid alkaloids was necessary for the manifestation of important analgesic activity [34]. The natural diterpenoid alkaloid 9-deoxylappaconitine showed significant analgesic activity that was superior to the reference drug **1** [35]. The synthesis and anti-inflammatory activity of a series of lappaconitine derivatives with various substituents (including N-acyl derivatives) on the *N*-20 position were reported [36]. The anthranilic acid substituent on the C-4 position in aconitum alkaloids also had a noticeable effect on the pharmacological activity. Modification at the nitrogen atom on the anthranilic acid moiety of *N*-deacetyllappaconitine **2** caused obvious changes in the analgesic activity and toxicity of the native alkaloid [37]. Our group previously described the modification of lappaconitine **1** on the C-5′ position at the aromatic moiety, which reduced the toxicity of the native alkaloid, enhanced its antiarrhythmic properties [38,39], and showed some marked analgesic effects [40].

In this work, we reported the synthesis of lappaconitine derivatives containing 3*H*-1,5-benzodiazepine fragments on the C-5′ position of the aromatic ring. The 1,5-benzodiazepines are of particular interest with respect to their application in the field of drugs and pharmaceuticals. These compounds have been extensively used as anticonvulsant, sedative, and analgesic agents. They act as positive allosteric modulators of the GABA_A_ receptor complex present in neural tissue. Likewise, 3*H*-1,5-benzodiazepines have emerged as powerful pharmacophores [41,42]. We designed a modern and convenient synthetic approach to lappaconitine–1,5-benzodiazepine hybrids based on the acid-catalyzed reaction of *o*-phenylenediamine with in situ-formed lappaconitine alkynyl ketones. The antinociceptive and antiarrhythmic activities of the new type of hybrid compounds compared to lappaconitine **1** were also studied and discussed.

## 2. Results and Discussion

### 2.1. Chemical Synthesis

**Scheme 1:**
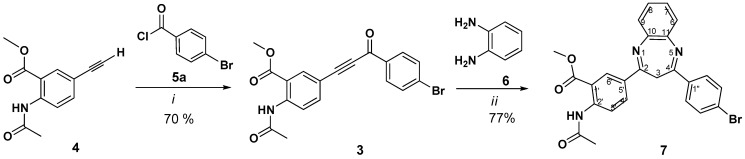
Synthesis of 2,4-diaryl substituted 3*H*-1,5-benzodiazepines. Reagents and conditions: (i): Pd(PPh_3_)_2_Cl_2_, PPh_3_, CuI, Et_3_N, C_6_H_6_, 65 °C, 8 h; (ii): MeCN, AcOH, reflux, 18 h.

In recent years, numerous attempts have been made toward the synthesis of 1,5-benzodiazepines from available nitrogen sources, under mild reaction conditions, and more facile operational procedures, including a multicomponent reaction strategy [43,44,45]. It was known that 1,3-diarylprop-2-yn-1-ones were reactive towards cyclocondensation with binucleophiles, including benzene-1,2-diamines [43]. These processes suffer from one or other limitations, such as requiring harsh conditions, expensive reagents, low or moderate yields, relatively long reaction times, and the occurrence of several side reactions. As a model reaction for synthesis of lappaconitine-3*H*-1,5-benzodiazepine hybrids, which are exemplified in the present article, we studied the synthesis of 1,3-diarylprop-2-yn-1-one **3** by the acyl Sonogashira reaction of methyl 2-(*N*-acetylamino)-5-ethynylbenzoate **4** [46] with 4-bromobenzoyl chloride **5a**, in previously described conditions [47,48], and the cyclocondensation reaction of alkynyl ketone **3** with *o*-phenylenediamine **6** (Scheme 1). For the last step, the choice of acetic acid has been beneficial to facilitate the cyclization step of the non-cyclic intermediate—Michael addition product. When the cyclocondensation reaction of **3** with *o*-phenylenediamine **6** was carried out in MeCN in the presence of acetic acid (6 equiv.) the substituted 3*H*-1,5-benzodiazepine **7** was obtained as the main product (yield 77%).

**Scheme 2:**
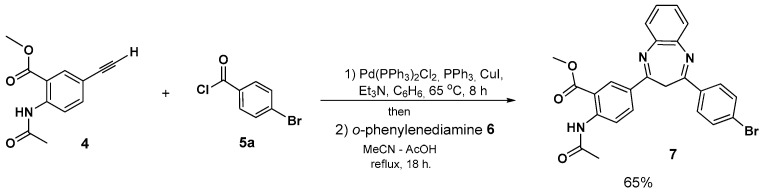
One-pot three-component synthesis of 2,4-diaryl substituted 3*H*-1,5-benzodiazepine.

For providing the most powerful approach to compound **7**, we assumed that two-step Pd-catalyzed Sonogashira coupling and subsequent Michael addition/cyclocondensation sequence could be carried out in a one-pot manner. Through the Sonogashira reaction of methyl *N*-acetyl-5-ethynylantranilate **4** with 4-bromobenzoyl chloride **5a** in benzene for 6 h (TLC-control), evaporation of the solvent, and a subsequent reaction of the crude alkynone with *o*-phenylene diamine **6** in MeCN in the presence of acetic acid, compound **7** was afforded in a yield of 65% (Scheme 2).

**Scheme 3:**
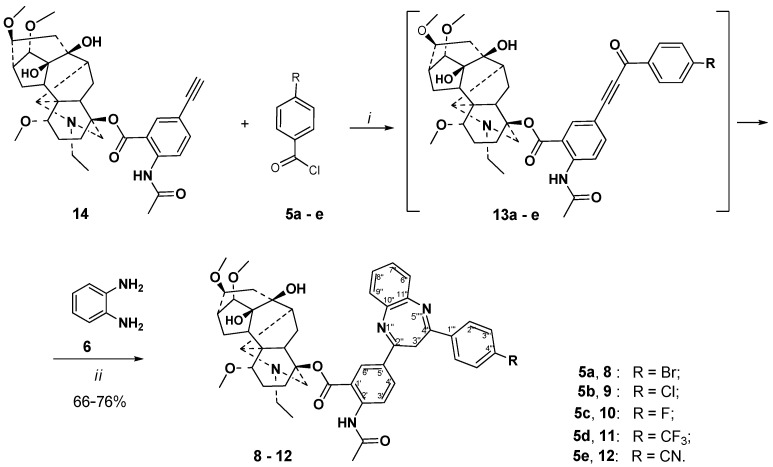
Alkaloid lappaconitine 1,5-benzodiazepine hybrids **8**–**12**. Reagents and conditions: (i): Pd(PPh_3_)_2_Cl_2_, PPh_3_, CuI, Et_3_N, C_6_H_6_, 65 °C, 8 h; (ii): MeCN, AcOH, reflux, 18 h.

We exploited this consecutive one-pot, three-component Sonogashira coupling/Michael addition/cyclocondensation sequence for the synthesis of lappaconitine–1,3-benzodiazepines **8**–**12**. The approach started with the catalytic generation of lappaconitine alkynyl ketones **13a**–**e**, which were easily accessible through acyl Sonogashira cross-coupling reaction of 5′-ethynyllappaconitine **14** [49] with benzoyl chlorides **5a**–**e**, in the previously found conditions [40]. The subsequent cyclocondensation reaction of the crude α,β-alkynyl ketones **13a**–**e** with *o*-phenylenediamine **6**, in the above found conditions, afforded 3*H*-1,5-benzodiazepine derivatives **8**–**12** with yields of 66–76%, through a two-stage process (Scheme 3).

**Figure 2:**
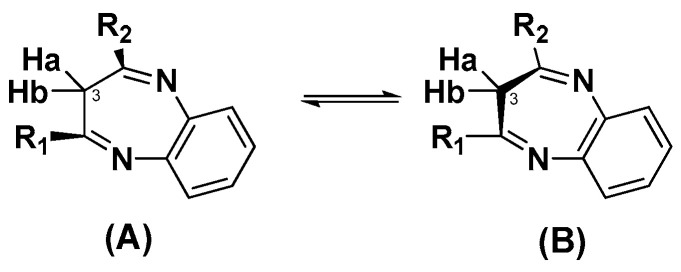
Conformational interconversion in structures **7** and **8**–**12**.

The structure of all synthesized compounds was established by NMR spectroscopy and mass spectrometry data. The ^1^H, and ^13^C NMR spectra of compounds **7** and **8**–**12** (recorded in CDCl_3_) were in good agreement with their structure and contained one set of characteristic signals of the 3H-1,5-benzodiazepine core, and the corresponding substituent (methyl anthranilate residue or lappaconitine fragment) (Supplementary Figures S2–S7). The ^1^H NMR spectra of 2,4-diaryl substituted 3*H*-1,5-benzodiazepine **7** have shown considerable line broadening for the methylene protons at C-3, which would be expected to be prone to rapid conformational interconversion of (A) and (B) (Figure 2) at an elevated temperature, leveling out the non-equivalence of the methylene protons. This dynamic process was studied by temperature-dependent NMR spectroscopy in (CD3)_2_SO between 293 and 363 K (Figures S2.2 and S2.3). It was revealed that as the recording of the temperature increased from 30 to 90 °C, a sharp singlet of CH2-protons at δ = 3.77 ppm gradually evolved (Figures S2.2 and S2.3). Characteristically, the carbon resonances of the C-3 methylene group appeared at δ = 34.44 ppm and evolved easily, assigned by the acquisition of the spectrum in CDCl_3_ at room temperature (Figure S2.4). Similarly, the CH_2_ protons at the C-3′ carbon atom appeared at a temperature of 333 K as a singlet at δ = 3.50 ppm (Figure S3), for compound **8** in (CD_3_)_2_SO, indicating an interconversion between A and B of the seven-membered rings (Figures S3.2 and S3.3). Early reports also described the interconversion between two structurally equivalent conformations (A) and (B) (Figure 2) [43,50]. Thus, the one-pot procedure for synthesized benzodiazepine derivatives in the tautomeric form of 3*H*-benzodiazepine from alkyne **14** was realized.

Therefore, we have developed a convenient synthetic route to hybrid compounds with lappaconitine and 3*H*-1,5-benzodiazepine structural fragments **8**–**12**. Next, we studied the analgesic and antiarrhythmic activity of new hybrid compounds.

### 2.2. Biological Study

#### 2.2.1. Analgesic Activity and Toxicity

The analgesic activity was studied by standard experimental pain models, namely, the acetic acid-induced writhing (0.75% acetic acid, 0.1 mL per 10 g, intraperitoneal) [51] and the hot plate (thermal stimulation, T = 54 °C) tests [52]. Agents were administered, intragastrically, 1 h before testing at doses of 5 mg/kg. Diclofenac sodium at the effective dose of 10 mg/kg was used as a reference drug. Lappaconitine **1** was used at the effective dose of 5 mg/kg. Table 1 presents the analgesic activity data of the novel benzodiazepine derivatives **7**, **8**, **9**, **10**, **11**, and **12** in the acetic acid-induced writhing test and hot plate test (oral administration). The hot plate test forms the basis for the study of analgesic activity and was used to detect the suppression of somatically superficial and acute pain. The acetic acid writhing test was the model for visceral pain, which indicates the involvement of the central nervous system in the analgesic response [53].

As it can be noted from the presented data, only the lappaconitine–benzodiazepine hybrid compound **8** exhibited significant analgesic activities in both experimental models: acetic acid-induced writhing test and hot plate test at the dose of 5 mg/kg.

Moreover, the activity of compound **8** at a dose of 5 mg/kg was comparable to the diclofenac sodium, administered at a dose of 10 mg/kg (Table 1). Lappaconitine **1** also exhibited a comparable statistically significant analgesic effect. Interestingly, other lappaconitine derivatives (**9**, **10**, **11**, and **12**) were inactive in both tests.

The 2,4-diarylsubstituted-3*H*-1,5-benzodiazepine **7** at a dose of 5 mg/kg showed a statistically significant decrease in the number of writhes. Yet, compound **7** was significantly less effective than the subsequent lappaconitine–benzodiazepine hybrid **8** in this test (pain inhibition of 21% and 54%, respectively). Additionally, compound **7** was inactive in the experimental model of thermal pain (hot plate test). It is worth noting that substances that showed analgesic activity in screening by the acetic acid writhing test are likely to mediate their analgesic response through the central nervous system by involving various neurotransmitter structures [53]

The analgesic activity of the lappaconitine–benzodiazepine hybrid **8** at a dose of 5 mg/kg was comparable to lappaconitine **1**. Therefore, we compared the activity of compound **8** after administration at doses of 1.0 and 5.0 mg/kg in both tests. The data is presented in Table 2. When administered intragastrically at a dose of 1 mg/kg compound **8** retained its effect (pain inhibition of 44% in the acetic acid-induced writhing test and 35% in the hot plate test). At the same time, at a dose of 5 mg/kg, its effect is somewhat more pronounced (Table 2).

Considering the fact, that lappaconitine–benzodiazepine hybrid **8** showed a significant analgesic effect in both pain tests, it is most likely that the nature of the substituent in the 4 position of the benzodiazepine ring has an important role in this class of hybrid compounds. It can be noted that the 4-bromophenyl substituent in the 4 position of the benzodiazepine ring system was necessary for the manifestation of the analgesic activity. The data obtained showed that 2-(4-N-acetylamino)-3-metoxycarbonylphenyl)-4-(4-bromophenyl)benzo-diazepine **7**, itself, also has an analgesic effect, although the replacement of (4-(*N*-acetylamino)-3-metoxycarbonylphenyl) moiety with the 4β-[2-(*N*-acetylamino)benzoyloxy]-8,9-dihydroxy-1α,14α,16β-trimethoxy-20-ethyl-18-noraconitane) substituent significantly enhanced the aforementioned activity.

Hybrid compound **8** was tested for acute toxicity in CD-1 mice using a single intragastric administration, according to the Kerber method. Compound **8** appeared to be moderately toxic with its LD_50_ exceeding the 1500 mg/kg value (oral administration). Thus, the toxicity of compound **8** was 50 times lower than the reference drug lappaconitine **1**. From these and our previously obtained data [40], it was evident that modification at the C-5′ position on the anthranilate moiety resulted in reducing the toxicity of C_18_–diterpenoid alkaloids.

#### 2.2.2. Studying the Antiarrhythmic Activity of Compounds **8** and **10**

**Figure 3:**
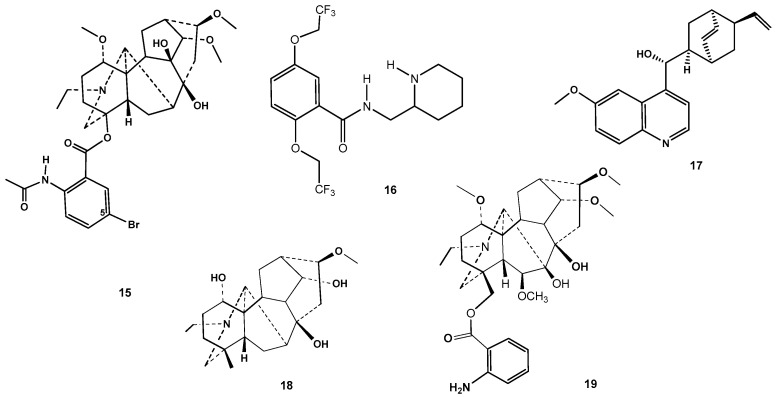
Structures of 5′-bromolappaconitine **15**, antiarrhythmic drug flecainide **16**, alkaloid quinidine **17**, and diterpenoid alkaloids karakoline **18** and anthranoyllycoctonine **19**.

The cardiotoxicity of lappaconitine **1** and *N*-deacetyllappaconitine **2** was much lower than aconitine, while lappaconitine **1** is a naturally occurring compound with class-I antiarrhythmic action [11]. This classification system of antiarrhythmics is based on in vitro studies of the agents’ electrophysiological effects. Antiarrhythmics of class I action are characterized by the slowing of conduction by Na^+^ channel blockade, with a slight effect on repolarization [11]. Along with the rather high clinical efficacy, lappaconitine hydrobromide (drug Allapinine), similar to other class 1 antiarrhythmics, produces serious side effects, including proarrhythmic action [13,17]. Lappaconitine **1** irreversibly blocks open human heart Na^+^ channels, which is in accordance with its antiarrhythmic activity [15,25]. Previously, it was found that common structural elements of effective antiarrhythmics included the presence of a residue of acetylanthranilic or anthranilic acid on C-4, methoxy groups on C-1, C-14, and C-16, and an OH on C-8 [7]. A substituent in the C-5′ position of the anthranylic acid moiety does seem to be crucial, although in this group, for example, 5′-bromolappaconitine **15** (Figure 3) was found to be a more potent antiarrhythmic than lappaconitine **1** [38,39]. The results of the electrocardiogram (ECG) parameters evaluation after the administration of agents **8** and **10** at a dose of 5 mg/kg are provided in Table S1 and Figures S8 and S9. The results revealed that agents **8** and **10** did not have a significant effect on the main parameters of the ECG (dR, dT, dP, QRS, ST, QT, Ra, Ta, and Pa).

##### The Antiarrhythmic Effect on In Vivo Models of Calcium Chloride and Epinephrine-Induced Arrhythmia

We studied the antiarrhythmic efficacy of lappaconitine–benzodiazepine hybrids **8** and **10** in two induced arrhythmia models in vivo. The results of this study are presented in Table 3. Studies of the antiarrhythmic effect on a model of calcium chloride-induced arrhythmia were conducted at a dose of 5 mg/kg. During the study, none of the two agents showed antiarrhythmic activity in this model. Increasing the dose of compounds **8** or **10** from 5 to 10 mg/kg led to 100% death of the animals (Figure S10). According to the data obtained, it can be assumed that the novel lappaconitine derivatives **8** and **10** do not affect the ionic activity of the cell membrane of cardiomyocytes associated with the passage of Ca^2+^ ions.

While studying the antiarrhythmic action in a model of epinephrine-induced arrhythmia at a dose of 5 mg/kg, compound **8** was found to show a good antiarrhythmic effect, whereby a complete recovery of the rats’ ECG was observed (Table 3, Figure S11). Using compound **8** at a dose of 2.5 mg/kg in this type of arrhythmia led to a complete recovery of the ECG in 50% of cases. Reducing the dose to 1 mg/kg led to the death of the animals (Table 3). Compound **10** was found to exhibit a strong and selective antiarrhythmic effect in the epinephrine-induced arrhythmia model at a dose of 5.0 and 0.5 mg/kg. At a dose of 0.05 mg/kg, against the background of epinephrine arrhythmia, this compound promoted ECG recovery in 50% of the animals (Table 3).

Therefore, the results of the in vivo experiments showed that lappaconitine–1,5-benzodiazepine hybrids with the fluorine or bromine substituent in 4-aryl moiety of the benzodiazepine core, **8** and **10**, do not prevent calcium chloride arrhythmia at the dosage found for lappaconitine hydrobromide (2.9 mg/kg). The blocking of epinephrine arrhythmia by the new derivatives of lappaconitine occurred when they were administered at a lower dose. This is especially important for compound **10**, which fully prevented the epinephrine arrhythmia at a dose of 0.5 mg/kg (a dosage six times lower than for lappaconitine hydrobromide. Thus, the new hybrid compounds were characterized as selective antiarrhythmics on epinephrine arrhythmia. Additionally, the data obtained have shown that novel lappaconitine derivatives **8** and **10** could affect cardiomyocyte adrenoceptors, the blockade of which led to a pronounced antiarrhythmic effect on the arrhythmia induction by the administration of a lethal dose of epinephrine.

##### Ex Vivo Research

The antiarrhythmic effects of agents commonly used in clinical medicine appear by blocking sodium [25,54], potassium [55], and calcium ion [56] channels. The traditional view is that lappaconitine **1** blocks the voltage-gated sodium channels by binding to sodium channel site 2 and reducing the Na^+^ inward flow, thus, further blocking K^+^ inward flow and influencing the inhibition of action and the potential generation and slowing the onset of pain [15].

To obtain more detailed information about the mechanism of the antiarrhythmic action of lappaconitine derivatives **8** and **10**, a study on the effect of the contraction of the isolated right atrium of rats was undertaken. At the beginning of this experiment, a preliminary study of the effect of arrhythmogens on the atrial contraction was conducted (Supplementary Tables S1–S3). In Figure S12 and Table S2, the effects of epinephrine, barium chloride, and calcium chloride on the contractions of the isolated rat atrium are shown. The preliminary results showed that arrhythmogens significantly changed the amplitude and slightly increased the frequency of contractions in the case of epinephrine.

Further, compound **8** or **10** was added to the cuvette with the isolated atrium at a concentration of 10^−3^ M. This concentration was selected as the working concentration from 10^−3^, 10^−4^, 10^−5^, and 10^−6^ M. Based on the data obtained, it was found that when epinephrine or barium chloride were administrated against the background of compounds **8** or **10**, atrial contractions did not significantly change their amplitude, although their frequency was slightly increased, which was less of a change than the ones that occurred when only epinephrine was administered. However, changes in atrium contractions against the background of both compounds **8** and **10** were observed for calcium chloride administration. Therefore, with the introduction of epinephrine and barium chloride against the background of compounds **8** or **10**, there was a relief of changes in the contraction of the sinus mode. Pathological contractions caused by the introduction of calcium chloride against the background of **8** or **10**, clearly showed the absence of the studied agents’ influence on the calcium channels of cardiomyocytes. This data was in agreement with the effect obtained by experiments in vivo (Table 3). Overall, according to the studies on the isolated atrium, it can be assumed that the synthesized hybrid compounds **8** and **10** act through the mechanisms of potassium channels and beta-adrenergic receptors blockade.

### 2.3. Molecular Modeling of a Possible Mechanism of Antinociceptive and Antiarrhythmic Potency of Lappaconitine-1,5-Benzodiazepine Hybrids ***8*** and ***10***

Regarding the mechanism of antinociceptive action of a variety of alkaloids with diterpenoid skeletons, the inhibition of the voltage-dependent Na^+^ channels and the blocking of the delayed rectifier K^+^ current were the key components. The latter may play a role in the antiarrhythmic effect because voltage-gated K^+^ channels have a crucial role in the regulation of the heart rate. Several studies have indicated that β-adrenoreceptor antagonists reduced, while the agonists enhanced, the central analgesic effect of diterpenoid alkaloids [9,10].

It is worth noting, there was no uniformity in the molecular mechanism of analgesia by lappaconitine **1**. The analgesic activity of lappaconitine **1** could be explained by the inhibition of NaV1.3, NaV1.4, NaV1.5, NaV1.7, and NaV1.8 voltage-gated sodium channels (VGSCs) [15,18]. Lappaconitine has been shown to irreversibly block NaV1.5 channels; however, channels with lysine substitutions within the local anesthetic receptor region at residue F1760 or N1765 are resistant to being blocked by lappaconitine [15]. These data suggest that by introducing a positive charge within the vicinity of the local anesthetic binding site, a disruption of lappaconitine binding to NaV1.5 channels occurs. NaV1.5 α-subunits have been associated with cardiac channelopathies [57]. It is well established that VGSCs regulate excitability in nociceptive neurons, and they become dysregulated in pain states [58,59].

VGSCs NaV1.5 is distinguished from other sodium channels by a unique glycosyl moiety and loss of disulfide-bonding capability at the NaV β subunit-interaction sites [60]. Class I antiarrhythmic drugs terminate and prevent cardiac arrhythmia by blocking cardiac sodium channels in a complex state-dependent manner [61]. The class 1C antiarrhythmic drug flecainide [*N-*(piperidin-2-ylmethyl)-2,5-bis(2,2,2-trifluoroethoxy)benz-amide] **16** (Figure 3) specifically targets the central cavity of the sodium channel pore. Flecainide **16** binds in the central cavity of NaV1.5, just on the intracellular side of the selectivity filter. Its piperidine ring lies across the top of the central cavity, hindering the exit of Na^+^ from the selectivity filter. The positively charged piperidine nitrogen points upward toward the inner exit from the selectivity filter and the hydrophobic edge of the piperidine ring extends towards the phenyl side chain of Phe1762 in the IVS6 segment. The wall of the central cavity of NaV channels is penetrated by four fenestrations that lead inward from the lipid bilayer between two pore modules. The two hydrophobic trifluoroethoxy tails of flecainide latch onto the inner end of the fenestration between the pore module in domain II and domain III (Figure S14).

Apparently, sodium channel blockers of various chemical classes occupy different positions within the channel pore, interacting with different amino acids. Allosteric regulation was also possible [62]. It is noted that flecainide **16** and quinidine **17** have distinct binding poses. Quinidine **17** is encaged completely within the central cavity, with the quinuclidine group directly cutting off the central permeation path and the quinolone double ring mainly coordinated by residues in domains III and IV. Quinidine binding triggers the rotation of Tyr1767, resulting in the rearrangement of the intracellular gate. The distinct chemical and structural properties of flecainide **16** and quinidine **17** and their different binding poses, thus, underlie their deviations in channel binding kinetics and impact on the modification of action potentials (Figure S14) [63]. Given these data, it should be taken into account that a direct comparison of the estimated values of the docking binding energies of chemical compounds of various classes and the inner surface of the sodium channel pore does not seem adequate.

According to electron microscopic models, flecainide **16** and quinidine **17** do not form hydrogen bonds and stacking interactions in the sodium channel pore (Figure S14A,C). A molecular dynamics study of flecainide in the sodium channel pore confirmed these data, showing the predominance of hydrophobic contacts and the low frequency of possible stacking interactions of the phenyl aromatic ring of flecainide [64]. The use of the induced fit docking method takes into account the conformational changes in the side chains of amino acids as a result of interaction with the ligand, thereby making it possible to obtain lower values of binding energy during modeling. In this case, the docking score for flecainide **16** was −9.432 kcal/mol, and for quinidine **17** it was −8.812 kcal/mol. In the case of flecainide **16**, stacking with the Phe1461 and Phe1420 π-systems can occur (Figure S14B), which was noted according to the results of a molecular dynamics study [64]. This kind of dynamic behavior by the sodium channel blocker molecule may reflect its “slow” kinetics. Apparently, rapid fluctuations with the formation of non-covalent interactions can also be expected from the quinidine molecule **17**, the nitrogen atom of the quinuclidine nucleus, which can be easily protonated with the subsequent formation of electrostatic interactions inside the pore of the sodium channel, as can be seen from the simulation using IFD (Figure S14D).

Induced fit docking results for lappaconitine **1**, its derivatives **8**, **10**, and **15**, and natural C-19 diterpenoid *Aconitum* alkaloids karacoline **18** [65] and anthranoyllycoctonine **19** [66] (Figure 3) are presented in Table 4.

Close IFD score values for all the compounds under consideration indicate identical conformational changes in the binding site of the sodium channel pore during interactions with these ligands. In this regard, the ability of the ligands to block the sodium channel pore is largely related to the energy of electrostatic and van der Waals interactions with the amino acids of the binding site and the internal energy of the considered ligand conformation. These energies are approximated using the docking score and Emodel parameters. Compound **10** possesses the most optimal combination of these parameters, slightly yielding to compound **8** in the docking score, but significantly exceeding it in terms of the Emodel parameter. The docking scores of lappaconitine **1** and 5′-bromolappaconitine **15** were significantly higher than the modified derivatives **8** and **10**. The difference between the Emodel parameters for all studied compounds is clearly visible. The presence of a more compact substituent containing only one aromatic ring in lappaconitine **1** and its derivative **15** or the absence of an aromatic substituent in native alkaloid **18** significantly increased the energy Emodel due to a smaller number of possible stacking and electrostatic interactions compared to semi-synthetic derivatives **8** and **10**. The decrease of the energy Emodel for the compound of the lycoctonine series **19** was observed. All these data confirm the positive effect of the larger substituent of the modified compounds on binding.

**Figure 4:**
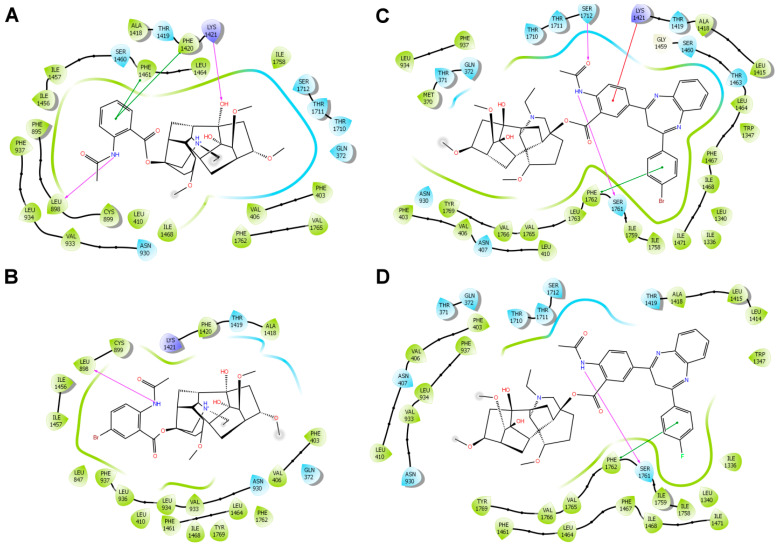
Diagrams of interactions of **1** (**A**), **15** (**B**), **8** (**C**), and **10** (**D**) with amino acids of the sodium channel pore as a result of IFD. Noncovalent interactions are shown with lines: hydrogen bonds are purple, electrostatic interactions are red, and stacking interactions are green.

The interaction diagrams for compounds **1**, **8**, **10**, and **15** are shown in Figure 4. The differences between compounds **8** and **10** at the binding site were not large. However, the presence of a bromine atom in the substituent of compound **8** (Figure 4C) can lead to the additional formation of noncovalent interactions with Ser1712 and Lys1421. Apparently, the length of the C–F bond in the substituent of compound **10** (Figure 4D) is significantly shorter than the C–Br bond of compound **8**, which leads to a deeper immersion of compound **10** into the pocket, moving the polar atoms away from the opposite side of the sodium channel pore. Lappaconitine **1** (Figure 4A) and 5′-bromolappaconitine **15** (Figure 4B) are located in the central part of the sodium channel pore. The presence of a bromine atom in structure **15** can probably create certain conformational difficulties in comparison with lappaconitine **1**, which manifests itself in a smaller number of noncovalent interactions. Apparently, the ability to penetrate between the fenestrations of the lipid membrane enhanced the binding of compounds within the pore, increasing their time of interaction with the sodium channel.

Compounds **8** and **10** blocked the sodium channel pore with the diterpenoid core. The lateral 3*H*-benzodiazepine substituent of the new compounds was located in the pocket between the fenestrations of the lipid membrane. The flecainide molecule **16** is located completely in the lumen of the sodium channel pore without interacting with fenestrations (Figure S15).

## 3. Materials and Methods

### 3.1. Chemistry

#### General Information

Melting points were determined using termosystem Mettler Toledo FP900 (USA). ^1^H NMR and ^13^C NMR spectra were recorded by using a Bruker AV 400 [400.13 (^1^H), 100.78 MHz (^13^C)] or DRX 500 [500.13 (^1^H), 125.77 MHz (^13^C)] spectrometer. Deuterochloroform (CDCl_3_) was used as a solvent, with residual CHCl_3_ (δ_H_ = 7.24 ppm) or CDCl_3_ (δ_C_ = 76.8 ppm) being employed as internal standards; by using (CD_3_)_2_SO as a solvent the residual of DMSO (δ_H_ = 2.51 ppm) was employed as an internal standard. Chemical shifts are provided in ppm and coupling constants (*J*) are presented in Hz. In the description of the NMR spectra of all new derivatives the core atom numbering denoted in structures **1** and **7** was used. Signals in the NMR ^1^H and ^13^C spectra of the diterpene alkaloid part of new compounds were assigned by correlation with those of lappaconitine **1** [67]. Mass spectra were determined by a ThermoScientific DFS high-resolution mass spectrometer (evaporator temperature 200–250 °C, EI ionization at 70 eV). The reaction progress and the purity of the obtained compounds were monitored by TLC on Silufol UV–254 plates (Kavalier, Prague, Czech Republic), eluted with CH_2_Cl_2_ for **3** or CHCl_3_-EtOH, 20:1 for **7**; detection under UV light). Methyl *N*-acetyl-5-ethynylantranilate **4** [46] and 5′-ethynyllappaconitine **14** [49] were prepared using the reported methods. Other reagents were purchased from commercial sources and were used without further purification. Solvents (CH_3_CN, benzene, CHCl_3_, CH_2_Cl_2_, EtOH) AcOH, and Et_3_N were purified by standard methods. HPLC analyses were carried out using an HPLC-UV (Agilent 1100, Agilent Technologies Inc., Santa Clara, CA, USA) with a Zorbax Bonus RP column (150 mm × 4.6 mm with 5 µm particle size; Agilent Technologies Inc., USA). The injection volume was 10 µL, and the column was thermostatically controlled at 35 °C. The mobile phase was composed of A (water) and B (methanol) with the following gradient elution: 0 min—1% B; 20 min—100% B; 30 min—100% B, the flow rate was set to 1.0 mL/min, and peaks were detected using a wavelength of 240 nm.

All synthesized compounds were judged as >95% pure by HPLC (an example: HPLC-UV chromatogram for compound **8**, Figure S16).

### 3.2. Synthesis and Spectral Data

#### 3.2.1. Methyl 2-acetylamino-5-(3-(4-bromophenyl)propioloyl)benzoate (**3**)

A solution of methyl 2-(N-acetylamino)-5-ethynylbenzoate 4 [46] (217 mg, 1 mmol) in benzene (5 mL) was added under argon (drop by drop over 1 h) to a mixture of 4-bromobenzoyl chloride (263 mg, 1.2 mmol) 5a, Pd(PPh_3_)_2_Cl_2_ (7 mg, 0.01 mmol), PPh_3_ (4 mg, 0.018 mmol), CuI (4 mg, 0.02 mmol), and Et_3_N (300 mg, 3 mmol) in benzene (10 mL). The reaction mixture was heated under stirring at 65 °C (bath) for 8 h (TLC). After cooling, the precipitate was filtered and washed with benzene, the combined organic layer was washed with an aqueous 3% ammonia solution (10 mL), dried over anhydrous sodium sulfate, and filtered. The solvent was removed under reduced pressure, the solid residue was purified by column chromatography on silica gel (eluent CH_2_Cl_2_) to afford compound 3 (279 mg, yield 70%), as a white powder. Rf 0.5 (CHCl_3_). M.p. 163.4–164.2 °C. NMR ^1^H (300 MHz, CDCl_3_) δ 11.23 (s, 1H, CH_3_CONH) 8.81 (d, J = 8.9 Hz, 1H, CH-3), 8.36 (d, J = 1.9 Hz, 1H, CH-6), 8.04 (d, J = 8.7 Hz, 2H, CH-2′, 6′), 7.79 (dd, J = 8.9, 1.9 Hz, 1H, CH-4), 7.65 (d, J = 8.7 Hz, 2H, CH-3′, 5′), 3.96 (s, 3H, CH_3_OCO), 2.26 (s, 3H, CH_3_CONH). NMR ^13^C (125 MHz, CDCl_3_), δ, 176.5 (C-9), 169.2 (CH_3_CONH), 167.6 (CH_3_CH_2_OCO), 143.4 (C-2), 138.7 (C-4), 136.2 (C-6), 135.4 (C-1′), 131.9 (C-2′, 6′), 130.8 (C-3′, 5′), 129.5 (C-4′), 120.3 (C-3), 114.7 (C-1), 113.3 (C-5), 92.5 (C-7), 86.6 (C-8), 52.7 (CH_3_OCO), 25.5 (CH_3_CONH). HR-MS (ESI) *m*/*z* Calcd. for C_19_H_14_BrNO_4_ [M]^+^ 399.0101, Found 399.0102.

#### 3.2.2. Methyl 2-Acetylamino-5-(4-(4-bromophenyl)-3H-benzo[b][1,4]diazepin-2-yl)benzoate [2-(Methyl 2-acetylaminobenzoate-5-yl)-4-(4-bromophenyl)-3H-1,5-benzodiazepine] (**7**)

A stirred mixture of compound **3** (399 mg, 1 mmol), *o*-phenylenediamine **6** (124 mg, 1.15 mmol), and acetic acid (360 mg, 6 mmol) in MeCN (10 mL) was refluxed for 18 h. After cooling, a white precipitate was formed and filtered, washed with cooled MeCN (2 × 5 mL), collected, and dried in a vacuum, to afford compound **7** (376 mg, 77%). An analytical sample was obtained after column chromatography on silica gel as a white powder. M.p. 191.0 °C (decomp.). NMR ^1^H (500 MHz, CDCl_3_) δ: 11.17 (s, 1H, CH_3_CONH) 8.79 (d, *J* = 8.8 Hz, 1H, CH-3′), 8.54 (d, *J* = 2.2 Hz, 1H, CH-6′), 8.15 (dd, *J* = 8.8, 2.2 Hz, 1H, CH-4′), 7.82 (d, *J* = 8.7 Hz, 2H, CH-2″, 6″), 7.57 (m, 2H, CH-7, 8), 7.54 (d, *J* = 8.7 Hz, 2H, CH-3″, 5″), 7.33 (m, 2H, CH-6, 9), 3.91 (s, 3H, CH_3_OCO), 2.24 (s, 3H, CH_3_CONH). NMR ^1^H (400 MHz, (CD_3_)_2_SO), 90 °C) δ: 10.58 (s, 1H, CH_3_CONH), 8.52 (d, *J* = 2.1 Hz, 1H, CH-6′), 8.44 (d, *J* = 8.6 Hz, 1H, CH-3′), 8.35 (dd, *J* = 8.6, 2.1 Hz, 1H, CH-4′), 8.03 (d, *J* = 8.5 Hz, 2H, CH-2″, 6″), 7.67 (d, *J* = 8.5 Hz, 2H, CH-3″, 5″),7.55 (m, 2H, CH-7, 8), 7.38 (m, 2H, CH-6, 9), 3.91 (s, 3H, CH_3_OCO), 3.77 (br s, CH_2_), 2.16 (s, 3H, CH_3_CONH). ^13^C (125 MHz, CDCl_3_) δ: 169.2 (CH_3_CONH), 168.2 (CH_3_OCO), 152.3 (C-4 or C-2), 151.8 (C-2 or C-4), 143.2 (C-2′), 140.3 (C-10, C-11), 135.7 (C-1″), 133.7 (C-6′), 131.8 (C-2″, 6″), 130.92 (C-5′), 130.88 (C-4′), 129.6 (C-3″, 5″), 128.6 (C-6 or C-9), 128.5 (C-9 or C-6), 125.7 (C-7 or C-8), 125.5 (C-7 or C-8), 125.4 (C-4″), 120.1 (C-3′), 114.6 (C-1′), 52.5 (CH_3_OCO), 34.5 (C-3), 25.5 (CH_3_CONH). HR-MS (ESI) *m/z* Calcd. for C_25_H_20_BrN_3_O_3_ [M]^+•^ 489.0683, Found 489.0687.

#### 3.2.3. General Procedure for Preparation of Lappaconitine-1,5-benzodiazepene Hybrids **8**–**12**

A solution of 5′-ethynyllappaconitine **14** [49] (550 mg, 0.9 mmol) in benzene (10 mL) was added under argon (drop by drop over 1 h) to a mixture of aroyl chloride (1.8 mmol) **5a**–**e**, Pd(PPh_3_)_2_Cl_2_ (6 mg, 0.009 mmol), CuI (3 mg, 0.018 mmol), PPh_3_ (4 mg, 0.018 mmol), and Et_3_N (250 mg, 2.7 mmol) in benzene (20 mL). The reaction mixture was heated under stirring at 65 °C (bath) for 8 h (TLC). After cooling, the precipitate was filtered and washed with benzene (10 mL), and the combined organic layer was washed with an aqueous 3% ammonia solution (10 mL), dried over anhydrous sodium sulfate, and filtered. The solvent was evaporated in a vacuum to afford the crude compounds **13a**–**e**. An acetonitrile solution (15 mL) containing compounds **13a**–**e**, o-phenylenediamine **6** (126 mg, 1.17 mmol), and acetic acid (378mg, 6.3 mmol) was stirred under reflux for 18 h. After cooling, the solvent was evaporated in a vacuum and the residue was crystallized with ethanol (~20 mL) to yield compounds **8**–**12**. To obtain pure samples, additional recrystallization from aqueous ethanol (1:4, *v*/*v*) was carried out.

##### 4β-{2′-Acetylamino-5′-(4″-(4-bromophenyl)-3H-1,5-benzodiazepine-2″-yl)benzoate}-1α, 14α,16β-Trimethoxy-20-ethylaconitane-8,9-diol (**8**)

Yield 76%, m.p. 156.5–157.8 °C, yellow powder. NMR ^1^H (500 MHz, CDCl_3_) δ: 11.20 (s, 1H, CH_3_CONH) 8.75 (d, *J* = 8.9 Hz, 1H, CH-3′), 8.44 (s, 1H, CH-6′), 8.17 (d, *J* = 8.9 Hz, 1H, CH-4′), 7.78 (d, *J* = 8.5 Hz, 2H, CH-2‴, 6‴), 7.60 (d, *J* = 8.5 Hz, 2H, CH-3‴, 5‴), 7.57 (m, 2H, CH-7″, 8″), 7.33 (m, 2H, CH-6″, 9″), 3.58 (d, *J* = 11.8 Hz, 1H, CH-19α), 3.57 (s, 1H, OH), 3.44 (d, *J* = 4.5 Hz, 1H, CH-14), 3.39 (s, 3H, CH_3_OC-14), 3.30 (s, 3H, CH_3_OC-16), 3.29 (s, 3H, CH_3_OC-1), 3.28–3.32 (m, 1H, CH-16), 3.22 (dd, *J* = 10.2, *J* = 7.1 Hz, 1H, CH-1), 3.01 (s, 1H, CH-17), 2.78 (dd, *J* = 14.8, *J* = 7.4 Hz, 1H, CH_2_-6α), 2.13–2.62 (m, 15H, CH_3_CONH, CH_2_-2β, CH-7, CH_2_-2α, CH-13, CH_2_-15α, CH-5, CH_2_-12α, 21α, 19β, CH_2_-21β, OH, CH_2_-3α), 2.15 (dd, *J* = 12.4, *J* = 4.4, 1H, CH-10), 1.96–2.05 (m, 2H, CH_2_-12β, CH_2_-15β), 1.78 (t, *J* = 12.5 Hz, 1H, CH_2_-3β), 1.62 (dd, *J* = 14.8, *J* = 8.3 Hz, 1H, CH_2_-6β), 1.12 (t, *J* = 7.1, 3H, CH_3_-22). NMR ^1^H (400 MHz, (CD_3_)_2_SO), 80 °C) δ: 10.52 (s, 1H, CH_3_CONH), 8.48 (s, 1H, CH-6′), 8.40 (d, *J* = 8.9 Hz, 1H, CH-3′), 8.33 (d, *J* = 8.9 Hz, 1H, CH-4′), 7.99 (d, *J* = 8.5 Hz, 2H, CH-2‴, 6‴), 7.70 (d, *J* = 8.5 Hz, 2H, CH-3‴, 5‴), 7.54 (m, 2H, CH-7″, 8″), 7.38 (m, 2H, CH-6″, 9″), 4.36 (s, 1H, OH), 3.89 (s, 1H, OH), 3.75 (br s, 2H, CH_2_), 3.43 (d, *J* = 11.2 Hz, 1H, CH-19α), 3.35–3.06 (m, 12H, CH-14, CH_3_OC-14, CH_3_OC-16, CH_3_OC-1, CH-16, CH-1), 2.94 (s, 1H, CH-17), 2.78 (dd, *J* = 14.8, *J* = 7.4 Hz, 1H, CH_2_-6α), 2.69–1.81 (m, 19H, CH_3_CONH, CH_2_-2β, CH-7, CH_2_-2α, CH-13, CH_2_-15α, CH-5, CH_2_-12α, 21α, 19β, CH_2_-21β, OH, CH_2_-3α, CH-10, CH_2_-12β, CH_2_-15β, CH_2_-3β), 1.46 (dd, *J* = 14.8, *J* = 8.3 Hz, 1H, CH_2_-6β), 1.05 (t, *J* = 7.1, 3H, CH_3_-22). NMR ^13^C (125 MHz, CDCl_3_), δ: 169.0 (CH_3_CONH), 166.8 (OCO), 152.5 (C-4″ or C-2″), 151.3 (C-2″ or C-4″), 143.4 (C-2′), 140.3 (C-10″ or C-11″), 140.2 (C-10″ or C-11″), 136.0 (C-1‴), 133.9 (C-6′), 132.0 (C-2‴, 6‴), 130.44 (C-5′), 130.42 (C-4′), 129.4 (C-3‴, 5‴), 128.6 (C-6″ or C-9″), 128.5 (C-9″ or C-6″), 125.6 (C-7″ or C-8″), 125.4 (C-7″ or C-8″), 125.3 (C-4‴), 120.2 (C-3′), 115.4 (C-1′), 89.9 (CH-14), 85.3 (C-4), 84.0 (CH-1), 82.7 (CH-16), 78.4 (C-9), 75.4 (C-8), 61.3 (CH-17), 57.8 (CH_3_OC-14), 56.5 (CH_3_OC-1), 56.0 (CH_3_OC-16), 55.3 (CH_2_-19), 50.9 (C-11), 49.6 (CH-10), 48.8 (CH_2_-21), 48.3 (CH-5), 47.4 (CH-7), 44.7 (CH_2_-15), 36.1 (CH-13), 33.9 (C-3″), 31.7 (CH_2_-3), 26.6 (CH_2_-2), 26.1 (CH_2_-12), 25.5 (CH_3_CONH), 24.0 (CH_2_-6), 13.4 (CH_3_-22).

##### 4β-{2′-Acetylamino-5′-(4″-(4-chlorophenyl)-3H-1,5-benzodiazepine-2″-yl)benzoate}-1α, 14α,16β-Trimethoxy-20-ethylaconitane-8,9-diol (**9**)

Yield 76%, m.p. 169.7–170.4 °C, yellow powder. NMR ^1^H (500 MHz, CDCl_3_) δ: 11.20 (s, 1H, CH_3_CONH) 8.74 (d, *J* = 8.9 Hz, 1H, CH-3′), 8.45 (s, 1H, CH-6′), 8.16 (d, *J* = 8.9 Hz, 1H, CH-4′), 7.85 (d, *J* = 8.6 Hz, 2H, CH-2‴, 6‴), 7.57 (m, 2H, CH-7″, 8″), 7.44 (d, *J* = 8.6 Hz, 2H, CH-3‴, 5‴), 7.33 (m, 2H, CH-6″, 9″), 3.58 (d, *J* = 11.8 Hz, 1H, CH-19α), 3.57 (s, 1H, OH), 3.44 (d, *J* = 4.5 Hz, 1H, CH-14), 3.39 (s, 3H, CH_3_OC-14), 3.30 (s, 3H, CH_3_OC-16), 3.29 (s, 3H, CH_3_OC-1), 3.28–3.32 (m, 1H, CH-16), 3.22 (dd, *J* = 10.2, *J* = 7.1 Hz, 1H, CH-1), 3.01 (s, 1H, CH-17), 2.78 (dd, *J* = 14.8, *J* = 7.4 Hz, 1H, CH_2_-6α), 2.13–2.62 (m, 15H, CH_3_CONH, CH_2_-2β, CH-7, CH_2_-2α, CH-13, CH_2_-15α, CH-5, CH_2_-12α, 21α, 19β, CH_2_-21β, OH, CH_2_-3α), 2.15 (dd, *J* = 12.4, *J* = 4.4, 1H, CH-10), 1.96–2.05 (m, 2H, CH_2_-12β, CH_2_-15β), 1.78 (t, *J* = 12.5 Hz, 1H, CH_2_-3β), 1.62 (dd, *J* = 14.8, *J* = 8.3 Hz, 1H, CH_2_-6β), 1.12 (t, *J* = 7.1, 3H, CH_3_-22). NMR ^13^C (125 MHz, CDCl_3_) δ: 169.0 (CH_3_CONH), 166.7 (OCO), 152.5 (C-4″ or C-2″), 151.3 (C-2″ or C-4″), 143.4 (C-2′), 140.3 (C-10″ or C-11″), 140.2 (C-10″ or C-11″), 136.7 (C-4‴), 135.5 (C-1‴), 133.9 (C-6′), 130.44 (C-5′), 130.42 (C-4′), 129.2 (C-2‴, 6‴), 129.0 (C-3‴, 5‴), 128.6 (C-6″ or C-9″), 128.5 (C-9″ or C-6″), 125.6 (C-7″ or C-8″), 125.4 (C-7″ or C-8″), 120.2 (C-3′), 115.4 (C-1′), 89.9 (CH-14), 85.3 (C-4), 84.0 (CH-1), 82.7 (CH-16), 78.4 (C-9), 75.4 (C-8), 61.3 (CH-17), 57.8 (CH_3_OC-14), 56.5 (CH_3_OC-1), 56.0 (CH_3_OC-16), 55.3 (CH_2_-19), 50.9 (C-11), 49.6 (CH-10), 48.8 (CH_2_-21), 48.3 (CH-5), 47.4 (CH-7), 44.7 (CH_2_-15), 36.1 (CH-13), 33.9 (C-3″), 31.7 (CH_2_-3), 26.6 (CH_2_-2), 26.1 (CH_2_-12), 25.5 (CH_3_CONH), 24.0 (CH_2_-6), 13.4 (CH_3_-22).

##### 4β-{2′-Acetylamino-5′-(4″-(4-fluorophenyl)-3H-1,5-benzodiazepine-2″-yl)benzoate}-1α, 14α,16β-Trimethoxy-20-ethylaconitane-8,9-diol (**10**)

Yield 76%, m.p. 223.8 °C (decomp.), yellow powder. NMR ^1^H (500 MHz, CDCl_3_) δ 11.20 (s, 1H, CH_3_CONH) 8.74 (d, *J* = 9.0 Hz, 1H, CH-3′), 8.48 (s, 1H, CH-6′), 8.16 (d, *J* = 9.0 Hz, 1H, CH-4′), 7.92 (d, *J* = 8.7, 5.4 Hz, 2H, CH-2‴, 6‴), 7.56 (m, 2H, CH-7″, 8″), 7.32 (m, 2H, CH-6″, 9″), 7.60 (d, *J* = 8.5 Hz, 2H, CH-3‴, 5‴), 3.58 (d, *J* = 11.8 Hz, 1H, CH-19α), 3.57 (s, 1H, OH), 3.43 (d, *J* = 4.5 Hz, 1H, CH-14), 3.39 (s, 3H, CH_3_OC-14), 3.30 (s, 3H, CH_3_OC-16), 3.29 (s, 3H, CH_3_OC-1), 3.28–3.32 (m, 1H, CH-16), 3.20 (dd, *J* = 10.2, *J* = 7.1 Hz, 1H, CH-1), 3.01 (s, 1H, CH-17), 2.80 (dd, *J* = 14.8, *J* = 7.4 Hz, 1H, CH_2_-6α), 2.15–2.64 (m, 15H, CH_3_CONH, CH_2_-2β, CH-7, CH_2_-2α, CH-13, CH_2_-15α, CH-5, CH_2_-12α, 21α, 19β, CH_2_-21β, OH, CH_2_-3α), 2.12 (dd, *J* = 12.4, *J* = 4.4, 1H, CH-10), 1.96–2.05 (m, 2H, CH_2_-12β, CH_2_-15β), 1.79 (t, *J* = 12.5 Hz, 1H, CH_2_-3β), 1.63 (dd, *J* = 14.8, *J* = 8.3 Hz, 1H, CH_2_-6β), 1.12 (t, *J* = 7.1, 3H, CH_3_-22). NMR ^13^C (125 MHz, CDCl_3_), δ, 169.0 (CH_3_CONH), 166.8 (OCO), 164.1 (d, *J* = 251.6, C-4‴), 152.3 (C-4″ or C-2″), 151.3 (C-2″ or C-4″), 143.4 (C-2′), 140.4 (C-10″ or C-11″), 140.3 (C-10″ or C-11″), 134.0 (C-6′), 133.3 (d, *J* = 2.4, C-1‴), 130.51 (C-5′), 130.45 (C-4′), 130.1 (d, *J* = 8.8, C-2‴, 6‴), 128.7 (C-6″ or C-9″), 128.5 (C-9″ or C-6″), 125.4 (C-7″ or C-8″), 125.3 (C-7″ or C-8″), 120.2 (C-3′), 115.8 (d, *J* = 21.9, C-3‴, 5‴), 115.4 (C-1′), 89.9 (CH-14), 85.3 (C-4), 84.0 (CH-1), 82.7 (CH-16), 78.4 (C-9), 75.4 (C-8), 61.3 (CH-17), 57.8 (CH_3_OC-14), 56.4 (CH_3_OC-1), 56.0 (CH_3_OC-16), 55.3 (CH_2_-19), 50.9 (C-11), 49.7 (CH-10), 48.8 (CH_2_-21), 48.3 (CH-5), 47.4 (CH-7), 44.7 (CH_2_-15), 36.2 (CH-13), 33.9 (C-3″), 31.7 (CH_2_-3), 26.6 (CH_2_-2), 26.1 (CH_2_-12), 25.4 (CH_3_CONH), 24.0 (CH_2_-6), 13.4 (CH_3_-22).

##### 4β-{2′-Acetylamino-5′-(4″-(4-trifluoromethylphenyl)-3H-1,5-benzodiazepine-2″-yl)-benzoate}-1α, 14α,16β-Trimethoxy-20-ethylaconitane-8,9-diol (**11**)

Yield 76%, m.p. 170.0 °C (decomp.), yellow powder. NMR ^1^H (400 MHz, CDCl_3_) δ 11.20 (s, 1H, CH_3_CONH) 8.75 (d, *J* = 9.0 Hz, 1H, CH-3′), 8.43 (s, 1H, CH-6′), 8.21 (d, *J* = 9.0 Hz, 1H, CH-4′), 8.01 (d, *J* = 8.1 Hz, 2H, CH-2‴, 6‴), 7.75 (d, *J* = 8.1 Hz, 2H, CH-3‴, 5‴), 7.57 (m, 2H, CH-7″, 8″), 7.34 (m, 2H, CH-6″, 9″), 3.61 (s, 1H, OH), 3.59 (d, *J* = 11.8 Hz, 1H, CH-19α), 3.44 (d, *J* = 4.5 Hz, 1H, CH-14), 3.39 (s, 3H, CH_3_OC-14), 3.30 (s, 3H, CH_3_OC-16), 3.29 (s, 3H, CH_3_OC-1), 3.28–3.32 (m, 1H, CH-16), 3.18 (dd, *J* = 10.2, *J* = 7.1 Hz, 1H, CH-1), 3.01 (s, 1H, CH-17), 2.82 (dd, *J* = 14.8, *J* = 7.4 Hz, 1H, CH_2_-6α), 2.16–2.65 (m, 15H, CH_3_CONH, CH_2_-2β, CH-7, CH_2_-2α, CH-13, CH_2_-15α, CH-5, CH_2_-12α, 21α, 19β, CH_2_-21β, OH, CH_2_-3α), 2.13 (dd, *J* = 12.4, *J* = 4.4, 1H, CH-10), 1.96–2.05 (m, 2H, CH_2_-12β, CH_2_-15β), 1.72 (t, *J* = 12.5 Hz, 1H, CH_2_-3β), 1.63 (dd, *J* = 14.8, *J* = 8.3 Hz, 1H, CH_2_-6β), 1.11 (t, *J* = 7.1, 3H, CH_3_-22). NMR ^13^C (100 MHz, CDCl_3_), δ, 169.0 (CH_3_CONH), 166.7 (OCO), 152.2 (C-4″ or C-2″), 151.0 (C-2″ or C-4″), 143.5 (C-2′), 140.3 (C-10″ or C-11″), 140.2 (C-1‴), 140.0 (C-10″ or C-11″), 134.0 (C-6′), 131.8 (q, *J* = 32.5 Hz, C-4‴), 130.3 (C-4′), 130.2 (C-5′), 128.60 (C-6″ or C-9″), 128.58 (C-9″ or C-6″), 128.1 (C-2‴, 6‴), 125.84 (C-7″ or C-8″), 125.75 (q, *J* = 3.6 Hz, C-3‴, 5‴), 125.4 (C-7″ or C-8″), 123.3 (q, *J* = 272.5 Hz CF_3_), 120.3 (C-3′), 115.3 (C-1′), 89.9 (CH-14), 85.3 (C-4), 84.0 (CH-1), 82.7 (CH-16), 78.4 (C-9), 75.4 (C-8), 61.3 (CH-17), 57.8 (CH_3_OC-14), 56.5 (CH_3_OC-1), 56.0 (CH_3_OC-16), 55.3 (CH_2_-19), 50.9 (C-11), 49.6 (CH-10), 48.8 (CH_2_-21), 48.3 (CH-5), 47.4 (CH-7), 44.7 (CH_2_-15), 36.1 (CH-13), 33.9 (C-3″), 31.7 (CH_2_-3), 26.6 (CH_2_-2), 26.1 (CH_2_-12), 25.5 (CH_3_CONH), 24.0 (CH_2_-6), 13.4 (CH_3_-22).

##### 4β-{2′-Acetylamino-5′-(4″-(4-cyanophenyl)-3H-1,5-benzodiazepine-2″-yl)benzoate}-1α, 14α,16β-Trimethoxy-20-ethylaconitane-8,9-diol (**12**)

Yield 70%, m.p. 178.1 °C (decomp.), yellow powder. NMR ^1^H (400 MHz, CDCl_3_) δ 11.19 (s, 1H, CH_3_CONH) 8.76 (d, *J* = 8.9 Hz, 1H, CH-3′), 8.44 (d, *J* = 2.0 Hz, 1H, CH-6′), 8.19 (dd, *J* = 8.9, 2.0, Hz, 1H, CH-4′), 8.01 (d, *J* = 8.5 Hz, 2H, CH-2‴, 6‴), 7.77 (d, *J* = 8.5 Hz, 2H, CH-3‴, 5‴), 7.57 (m, 2H, CH-7″, 8″), 7.34 (m, 2H, CH-6″, 9″), 3.60 (d, *J* = 11.8 Hz, 1H, CH-19α), 3.57 (s, 1H, OH), 3.43 (d, *J* = 4.5 Hz, 1H, CH-14), 3.38 (s, 3H, CH_3_OC-14), 3.30 (s, 6H, CH_3_OC-16, CH_3_OC-1), 3.28–3.32 (m, 1H, CH-16), 3.21 (dd, *J* = 10.2, *J* = 7.1 Hz, 1H, CH-1), 3.02 (s, 1H, CH-17), 2.81 (dd, *J* = 14.8, *J* = 7.4 Hz, 1H, CH_2_-6α), 2.16–2.66 (m, 15H, CH_3_CONH, CH_2_-2β, CH-7, CH_2_-2α, CH-13, CH_2_-15α, CH-5, CH_2_-12α, 21α, 19β, CH_2_-21β, OH, CH_2_-3α), 2.13 (dd, *J* = 12.4, *J* = 4.4, 1H, CH-10), 1.96–2.05 (m, 2H, CH_2_-12β, CH_2_-15β), 1.75 (t, *J* = 12.5 Hz, 1H, CH_2_-3β), 1.62 (dd, *J* = 14.8, *J* = 8.3 Hz, 1H, CH_2_-6β), 1.12 (t, *J* = 7.1, 3H, CH_3_-22). NMR ^13^C (125 MHz, CDCl_3_), δ, 169.0 (CH_3_CONH), 166.6 (OCO), 151.4 (C-4″ or C-2″), 150.9 (C-2″ or C-4″), 143.6 (C-2′), 140.8 (C-10″ or C-11″), 140.4 (C-10″ or C-11″), 139.8 (C-1‴), 134.1 (C-6′), 132.5 (C-2‴, 6‴), 130.3 (C-4′), 130.1 (C-5′), 128.7 (C-6″ or C-9″), 128.6 (C-9″ or C-6″), 128.3 (C-3‴, 5‴), 126.1 (C-7″ or C-8″), 125.5 (C-7″ or C-8″), 120.3 (C-3′), 118.3 (CN), 115.3 (C-1′), 113.6 (C-4‴), 89.9 (CH-14), 85.3 (C-4), 84.0 (CH-1), 82.7 (CH-16), 78.4 (C-9), 75.4 (C-8), 61.3 (CH-17), 57.8 (CH_3_OC-14), 56.5 (CH_3_OC-1), 56.0 (CH_3_OC-16), 55.2 (CH_2_-19), 50.9 (C-11), 49.6 (CH-10), 48.8 (CH_2_-21), 48.5 (CH-5), 47.4 (CH-7), 44.7 (CH_2_-15), 36.1 (CH-13), 33.6 (C-3″), 31.7 (CH_2_-3), 26.6 (CH_2_-2), 26.1 (CH_2_-12), 25.5 (CH_3_CONH), 24.0 (CH_2_-6), 13.4 (CH_3_-22).

### 3.3. Biological Evaluation

#### 3.3.1. Animals

The study of analgesic activity was carried out on CD-1 mice (male), weighing 20–25 g, with 8 animals in each group (SPF-vivarium of the Institute of Cytology and Genetics of the Siberian Branch of the Russian Academy of Sciences). The study of antiarrhythmic activity was carried out on mature male rats of the Wistar strain weighing 190–220 g, aged 2–3 months, with 10 animals in each group (rats were obtained from the Institute of Cytology and Genetics, the Siberian Branch of the Russian Academy of Sciences (Novosibirsk, Russia)). Experimental animals were maintained at 22–25 °C on a 12 h light–dark cycle with food and water available ad libitum. All work with animals was performed in strict accordance with the laws of the Russian Federation, the decree of the Ministry of Health of the Russian Federation no. 199n of 4 January 2016, and Directive 2010/63/EU of the European Parliament and of the Council of the European Union of 22 September 2010 on the protection of animals used for scientific purposes. The experiments were approved by the Ethics Committee of the N.N. Vorozhtsov Institute of Organic Chemistry SB RAS (protocol № 2/2016 from 15 February 2016, protocol №. 8/2017 from 9 November 2017, protocol № P-13-012121-15 from 15 January 2021).

#### 3.3.2. Analgesic Tests

Agents were dissolved in distilled water with a drop of Tween-80 just before use and were administered per os, 1 h before testing. Distilled water with a drop of Tween-80 was administered per os in mice of the control group, 1 h before testing. Analgesic activity of test agents was assessed using the acetic acid-induced writhing test and hot plate test. In the acetic acid-induced writhing test the pain reaction was determined by the number of abdominal convulsions, recorded from the 5th to the 8th min following the acetic acid injection (0.75%, 0.1 mL/10 g body weight) [51]. The percentage of pain reaction inhibition was calculated according to the following equation: % inhibition = 100 × (A − B)/A, where A is the mean number of writhes in the control group, and B is the mean number of writhes in the test group. In the hot plate test animals were placed individually on a metallic plate (VWR Hotplate/Stirrer 725-HPS, New Paris, OH, USA) and warmed to 54 ± 0.5 °C until either licking of the hind paw or jumping [52]. The pain response time was recorded by a stopwatch and the animal was immediately taken away from the plate and put back into the cage. Statistical analysis was conducted in STATISTICA 7.0 program using the Mann–Whitney U Test to assess the significant (*p* < 0.05) differences. The data are presented as mean ± standard error of the mean (SE).

#### 3.3.3. Antiarrhythmic Activity

The rats were anesthetized with sodium thiopental (0.12 mg/kg) by intraperitoneal administration. After the induction of anesthesia, the animals were restrained, and an electrocardiogram (ECG) was recorded. For this purpose, three electrodes were affixed to the body of the rats. The electrodes were connected to an ECG amplifier: the V75-11 ECG Isolated Amplifier (Coulbourn Instruments; Holliston, MA, USA). It performs all the standard multi-lead ECG monitoring functions under the control of LabView 6.1 software. Left ventricular pressure levels were measured with a high-fidelity low-cost blood pressure transducer (V94-21, Coulbourn Instruments; USA) placed in the left ventricle via the right carotid artery. Arrhythmia was induced by either a one-step intravenous injection of a 10% CaCl_2_ solution at a dose of 250 mg/kg or an epinephrine injection at 0.3 mg/kg into rats anesthetized using sodium thiopental (0.12 mg/kg, intraperitoneally). These doses of arrhythmogenesis were lethal (100% lethal dose: LD_100_) for rats. Tested agents were mixed with Tween-80, the mixtures were diluted with physiological saline, and the resulting formulations were injected into the vena femoralis (the same site in all the rats). This route of administration allowed the blocking of the acute arrhythmia directly. After the induction of anesthesia, the animals were restrained, and an ECG was recorded via a second standard lead for 10 min. The duration of the RR, PQ, QRS, QT, and P wave intervals were estimated together with amplitudes of the P, T, and R waves. Statistical analysis was performed using the STATISTICA 10.0 software and the software developed at the Novosibirsk Institute of Organic Chemistry for calculating ECG parameters.

Right atria from Wistar rats were prepared as described previously [38]. Briefly, the animals were sacrificed by CO_2_ asphyxiation, and the right atria were quickly excised and immersed in Ringer–Locke’s solution (approximate concentrations in grams per liter: NaCl (9.0–9.5), KCl (0.20–0.42), CaCl_2_ (anhydrous, 0.20–0.24), NaHCO_3_ (0.1–0.3), and glucose (1.0–2.5)). The right atria with sinus cardiac pacemakers were dissected and mounted in isolated organ baths filled with 10 mL of Ringer–Locke’s solution maintained at 37 °C and aerated with 95% O_2_ and 5% CO_2_. To achieve a steady spontaneous tone level, an initial tension of 1 g was applied. Contractions were measured isometrically with a force-displacement transducer (PanLab s.l., Barcelona, Spain) and recorded by Isolated Organs Data Acquisition software (Protowin; Panlab Technology for Bioresearch, Barcelona, Spain). Tissues were allowed to stabilize for 20 min, whereas the bathing solution was exchanged at 5 min intervals. After the re-establishment of stable baseline tone, our compounds were added to the tissues (10^−6^ to 10^−3^ M).

### 3.4. Molecular Docking Study

All theoretical calculations were carried out using the software Schrodinger Small Molecule Drug Discovery Suite 2020-2 [68]. The geometric parameters of the protein were downloaded from Protein Data Bank [69]. Full-size rat cardiac sodium receptor NaV1.5 with flecainide [60] (PBD ID 6UZ0, resolution 3.24 Å) and human sodium receptor NaV1.5 with quinidine [63] (PDB ID 6LQA, resolution 3.3 Å) electron microscopic models were selected for molecular modeling. Model protein structures were prepared by adding and minimizing hydrogen atoms, adding missing amino acid side chains, restoring bond multiplicity, removing solvent molecules, and the entire structures were optimized in the OPLS4 force field [70]. The geometric parameters of the ligands were optimized using the OPLS4 force field, considering all possible conformations. For docking into cardiac sodium receptor NaV1.5 models, the binding sites of antiarrhythmic drugs flecainide and quinidine were chosen, located in the central cavity of Nav1.5.

Molecular docking was performed using Induced Fit Docking (IFD) protocol [71], based on Glide [72] and the Refinement module in Prime [73], which accurately predicts ligand binding modes and concomitant structural changes in the receptor. The following conditions were applied: flexible protein and ligand, 20 Å grid matrix size, and amino acids within 5 Å of the ligand were constrained to be optimized for ligand influence. Docking results were ranked by evaluating the following calculation parameters: docking score (based on GlideScore with penalties exclusion), ligand efficiency (LE, where the per-heavy-atom distribution of the scoring function is considered), the model energy value parameter (Emodel), including GlideScore value, the energy of unbound interactions and energy parameters spent on the formation of compound stacking in the binding site, and IFDscore, including GlideScore value and a fraction of the Prime energy, which reflects the result of the structural changes in the receptor.

## 4. Conclusions

A three-component synthesis of hybrid molecules containing the diterpenoid alkaloid lappaconitine and 3*H*-1,5-benzodiazepine fragments has been developed. The one-pot sequence of 5′-alkynone-lappaconitines formation from 5′-ethynyllappaconitine followed by subsequent Michael addition/cyclocondensation with *o*-phenylenediamine afforded lappaconitine-1,5-benzodiazepine hybrid compounds. The protocol provides mild reaction conditions, high yields of products, and operational simplicity to assemble complex structural entities in a single operation. A lead compound, which possessed analgesic activity in vivo and antiarrhythmic effects on the epinephrine arrhythmia model was revealed. The lead compound exhibited high analgesic activity in acetic acid-induced writhing and hot plate tests and provided lower acute toxicity than the parent compound lappaconitine. Studies on the isolated atrium showed that the possible mechanism of action of the lappaconitine–benzodiazepine hybrids was through the blockade of beta-adrenergic receptors and potassium channels. At the same time, it did not seem to block the calcium channels. In conclusion, our results provide a basis for future rational use of the mentioned lappaconitine modification strategy for reducing its side effects, while also retaining the high therapeutic activity.

## Figures and Tables

**Table 1:** Analgesic activity of compounds **1**, **7**, **8**, **9**, **10**, **11**, and **12** and diclofenac sodium.

Compound	Dose, mg/kg	Acetic Acid-Induced Writhing Test	Dose, mg/kg	Hot Plate Test
		Mean ± SD (MPE, %) ^a^		Mean ± SD (MPE, %) ^b^
**7**	5	9.3 ± 0.94 * (21)	5	6.8 ± 0.37
**8**	5	5.3 ± 0.62 * (54)	5	12.8 ± 1.21 * (62)
**9**	5	10.9 ± 1.01	5	10.0 ± 1.16
**10**	5	10.8 ± 1.66	5	8.4 ± 0.78
**11**	5	12.0 ± 0.98	5	8.9 ± 0.74
**12**	5	12.0 ± 1.00	5	8.9 ± 0.64
**1**	5	5.7 ± 1.25 * (49)	5	12.3 ± 1.13 * (58)
Diclofenac sodium	10	3.1 ± 0.47 * (72)	10	11.3 ± 0.49 * (43)
Control	-	11.3 ± 0.62	-	7.9 ± 0.51

^a^ For the acetic acid-induced writhing test (MPE) = % of pain inhibition = (t_control_ − t_exp_)/t_control_ × 100%; * *p* < 0.05 vs. Control. ^b^ For the hot plate test (MPE) = % of pain inhibitioh = (t_exp_ − t_control_)/t_control_ × 100%; * *p* < 0.05 vs. Control.

**Table 2:** Analgesic activity of compound **8** and diclofenac sodium at doses of 1 and 5 mg/kg.

Compound	Dose, mg/kg	Acetic Acid-Induced Writhing Test	Dose, mg/kg	Hot Plate Test
		Mean ± SD (MPE, %) ^a^		Mean ± SD (MPE, %) ^b^
**8**	5	4.9 ± 0.59 * (59)	5	11.8 ± 0.65 * (61)
**8**	1	6.6 ± 1.07 * (44)	1	9.6 ± 0.94 * (35)
Diclofenac sodium	10	4.3 ± 0.65 * (64)	10	11.4 ± 0.68 * (56)
Control	-	11.9 ± 0.51	-	7.3 ± 0.62

^a^ For the acetic acid-induced writhing test (MPE) = % of pain inhibition = (t_control_ − t_exp_)/t_control_ × 100%; * *p* < 0.05 vs. Control. ^b^ For the hot plate test (MPE) = % of pain inhibitioh = (t_exp_ − t_control_)/t_control_ × 100%; * *p* < 0.05 vs. Control.

**Table 3:** Dependence of the survival rates on the dose of injected compounds **8**, **10**, and lappaconitine hydrobromide (Allapinine, positive standard) (rats model).

Compound	Dose, mg/kg	Surviving Animals (%)		Compound	Dose, mg/kg	Surviving Animals (%)		Compound	Dose, mg/kg	Surviving Animals (%)	
		Calcium Chloride Arrhythmia (250 mg/kg)	Epinephrine Arrhythmia (0.3 mg/kg)			Calcium Chloride Arrhythmia (250 mg/kg)	Epinephrine Arrhythmia (0.3 mg/kg)			Calcium Chloride Arrhythmia (250 mg/kg)	Epinephrine Arrhythmia (0.3 mg/kg)
**8**	10	0	100	**10**	10	0	100	Allapinine	2.9	100	100
	5.0	100	100		5.0	100	100		0.29	50	50
	2.5	NT	50		0.5	NT	100		0.029	0	0
	1.0	NT	0		0.05	NT	50		0.0029	0	0

**Table 4:** Induced FID docking results for diterpenoid ligands.

Ligand	IFD Calculation Parameters, kcal/mol			
	Docking Score	Ligand Efficiency	Emodel	IFD Score
**1**	−9.903	−0.236	−74.681	−2133.29
**8**	−17.711	−0.295	−108.167	−2241.11
**10**	−16.595	−0.277	−137.495	−2239.37
**15**	−9.529	−0.222	−79.817	−2133.29
**18**	−9.547	−0.354	−43.472	−2223.73
**19**	−11.250	−0.268	−100.692	−2227.94

## Data Availability

Data are contained within the article.

## Supplementary Materials

The following supporting information can be downloaded at: https://www.mdpi.com/article/10.3390/molecules28104234/s1, Figures S1–S7: ^1^H and ^13^C NMR Spectra of new compounds **3**, **7**–**12**; Figures S8 and S9: Effect of compound **8** or **10** injection on ECG parameters; Figure S10: Changes in the rat ECG during the administration of compound **8** on a model of calcium chloride arrhythmia; Figures S11–S13: The effect of administration of arrhythmogen and tested compounds **8** and **10**; Figures S14 and S15: Diagrams of interactions and superpositions of sodium channel blockers; Figure S16: HPLC-UV spectrum for compounds **8**; Tables S1–S3: Influence of compounds **8** and **10** on ECG parameters.

## Sample Availability

Samples of the synthesized compounds may be available from the authors but only with the permission of the Novosibirsk Institute of Organic Chemistry.
