# Supplementary material for: Synthesis, Pharmacological Evaluation, and Molecular Modeling of Lappaconitine–1,5-Benzodiazepine Hybrids
Source: Molecules. 2023 May 22;28(10):4234. doi: 10.3390/molecules28104234 (PMC10223824; doi:10.3390/molecules28104234)
Supplement: Supplementary file 1 [file molecules-28-04234-s001.zip › molecules-2404341-supplementary.pdf]

Supporting Information

Synthesis, pharmacological evaluation, and molecular modeling of lappaconitine–1,5-benzodiazepine hybrids

Kirill P. Cheremnykh [1], Arkadiy O. Bryzgalov [1], Dmitry S. Baev [1], Sergey A. Borisov [1], Yulia S. Sotnikova [1], Victor A. Savelyev [1], Tatyana G. Tolstikova [1], Shamansur S. Sagdullaev [2] and Elvira E. Shults [1,*]

[1] N.N. Vorozhtsov Novosibirsk Institute of Organic Chemistry, Siberian Branch, Russian Academy of Sciences, Lavrentjev Avenue 9, 630090 Novosibirsk, Russian Federation; schultz@nioch.nsc.ru

[2] S.Yu. Yunusov Institute of the Chemistry of Plant Substances, Academy of Sciences of the Republic of Uzbekistan, Mirzo Ulugbek str. 77, 100170 Tashkent, Uzbekistan; plant_inst@icps.org.uz

* Correspondence: schultz@nioch.nsc.ru; Tel.: +7-383-3308533

Table of Contents

1. NMR Spectra of new compounds **3,7-12**……………………………………….................. S1- S7.

2. Effect of compound **8** or **10** injection (dose 5mg/kg) on ECG parameters ..…………….. S8,9

3. Changes in the rat ECG during administration of compound **8** on a model of calcium chloride arrhythmia……………………………………………………..S 10

4. Arrhythmia in rats caused by the introduction of epinephrine and restoration of the rhythm against the background of the administered compound **8** ………………….S 11

5. Rat atrium bead alteration after arrhythmogen administration………………………….S 12

6. Rat atrium bead alteration after administration of arrhythmogen against the background of compounds **8** or **10**…………………………………………………….S13

7. Table S1-S3. Influence of compounds **8** and **10** on ECG parameters……….………….P 15

8. Fig S14. Diagrams of interactions of flecainide **16** and quinidine **17**…………….…….P. 16

9. Fig S15. Superposition of sodium channel blockers……………………………….....P. 17

9. Fig S16. HPLC-UV spectrum for compounds **8**……………………………….……..P. 18

Figure S 1.1. ^{1}H NMR spectrum for compound 3 ($CDCl_3$, 400 MHz)

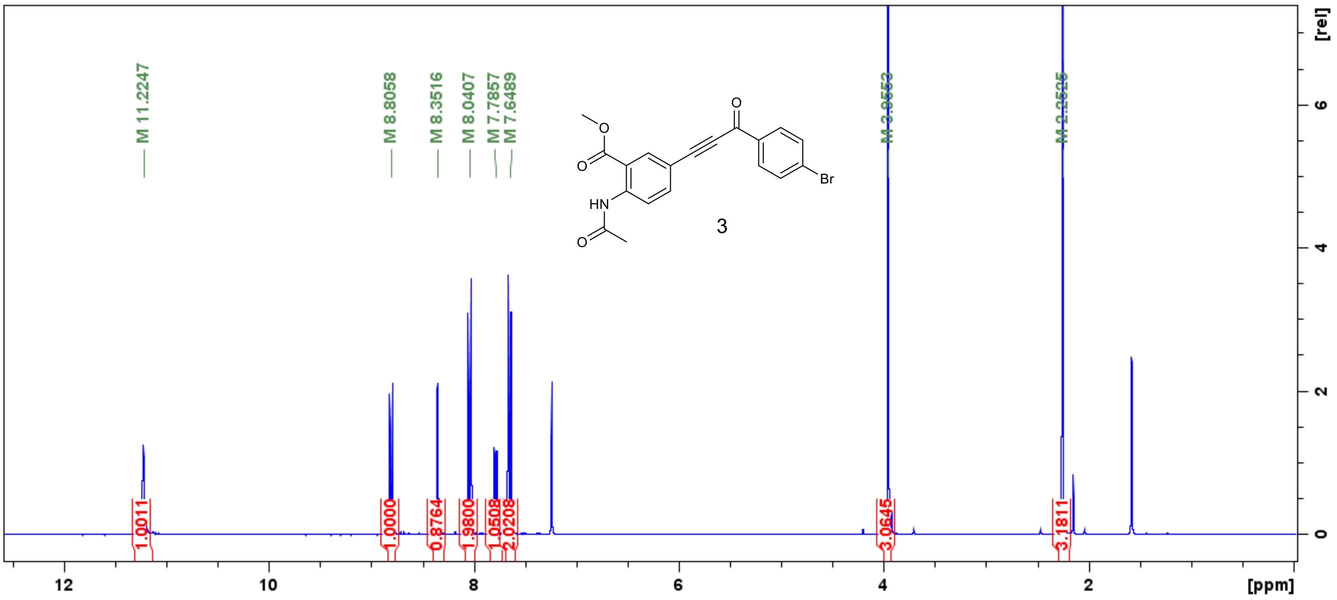

Figure S 1.2. **^{13}C NMR** spectrum for compound **3** ($CDCl_3$, 125 MHz)

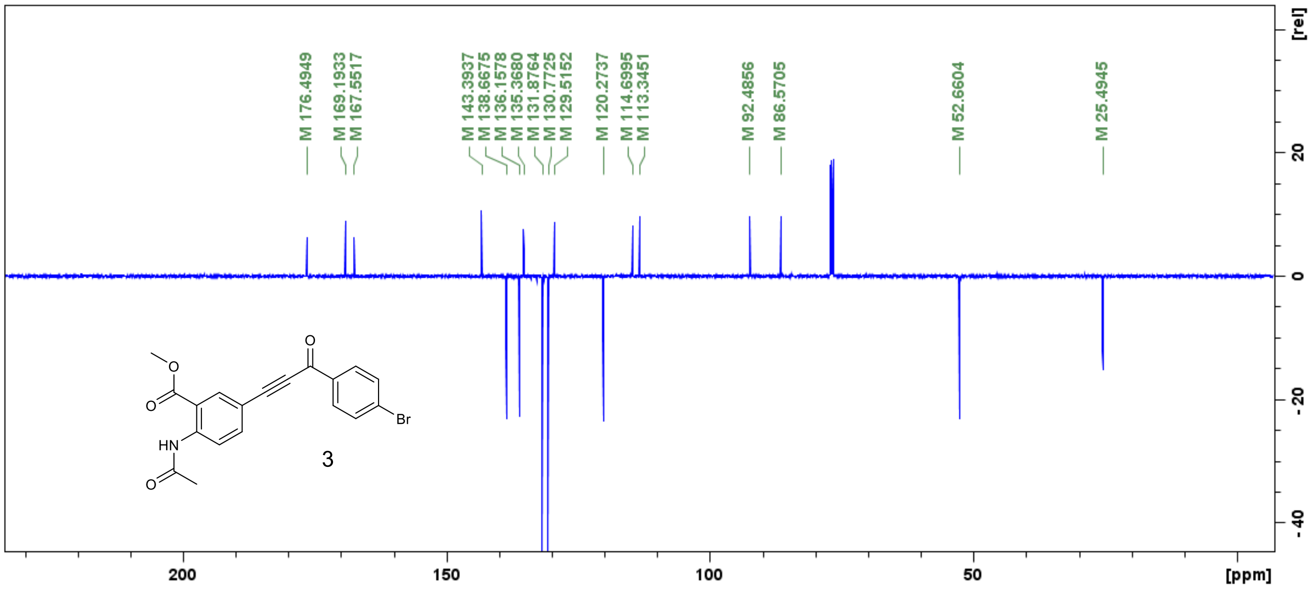

Figure S 2.1. ^{1}H NMR spectrum for compound **7** ($CDCl_3$, 500 MHz)

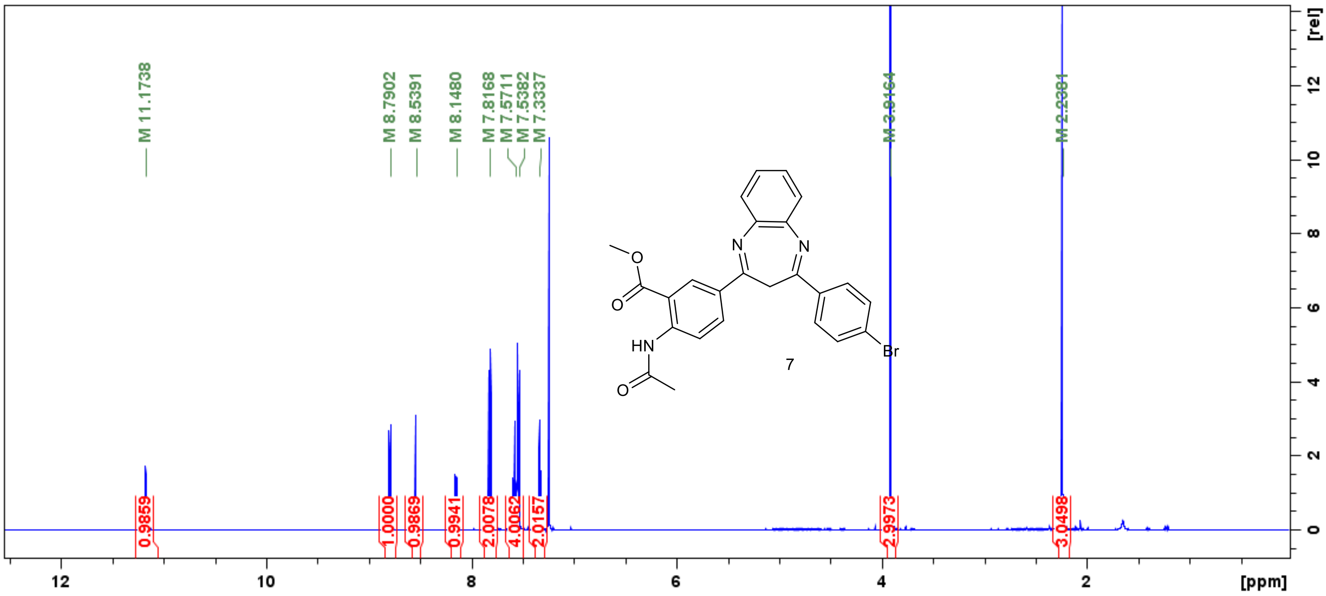

Figure S 2.2.Temperature dependent ^{1}H NMR spectrum for compound **7** recorded in $(CD_3)_2SO$, 400 MHz)

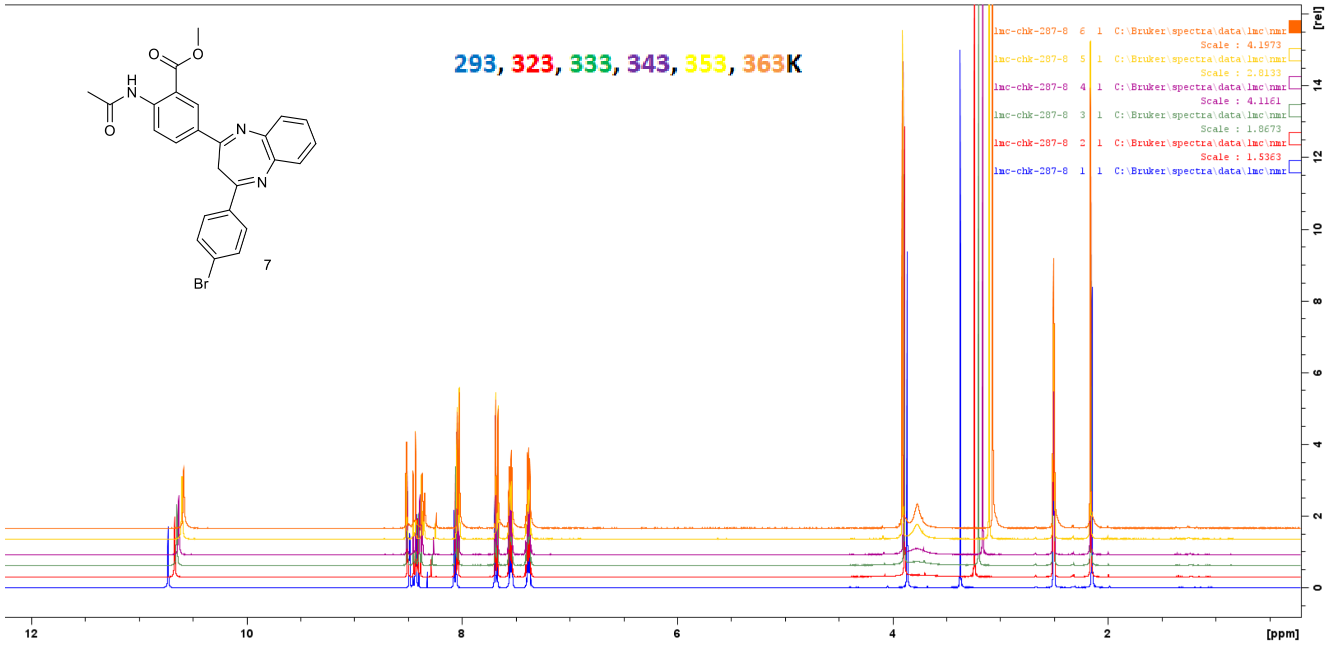

Figure S 2.3. Selected signals from spectra S2.2

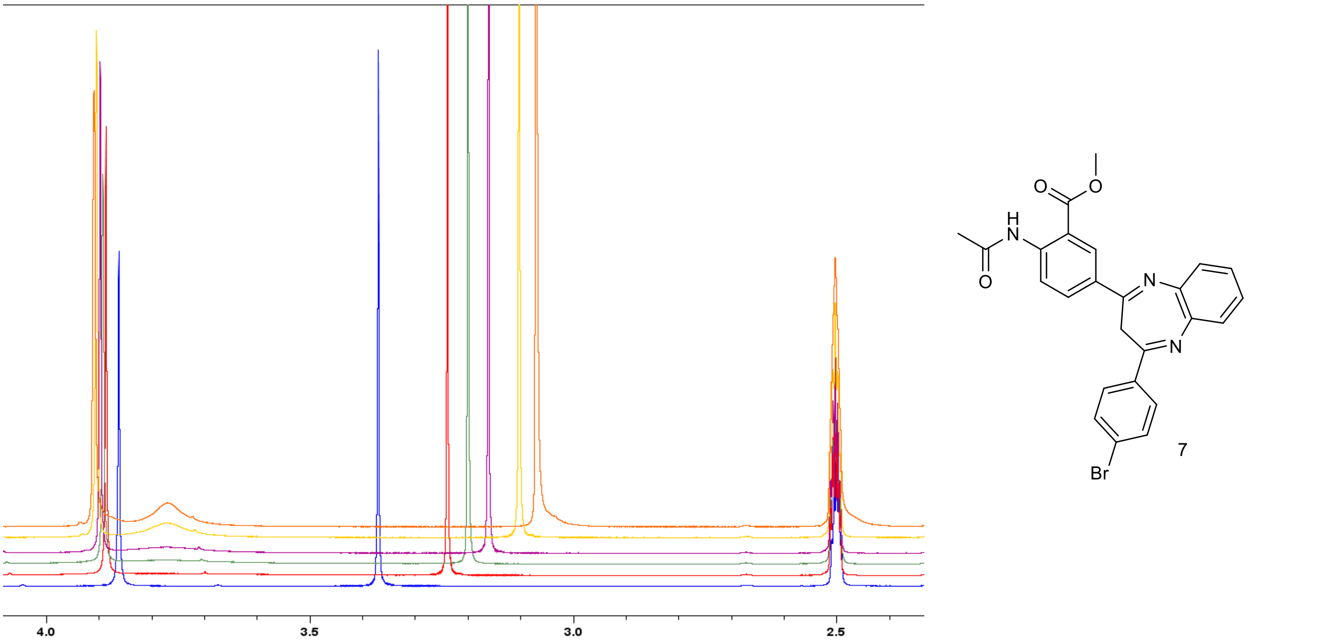

Figure S2.4. **^{13}C NMR** spectrum for compound **7** ($CDCl_3$, 125 MHz)

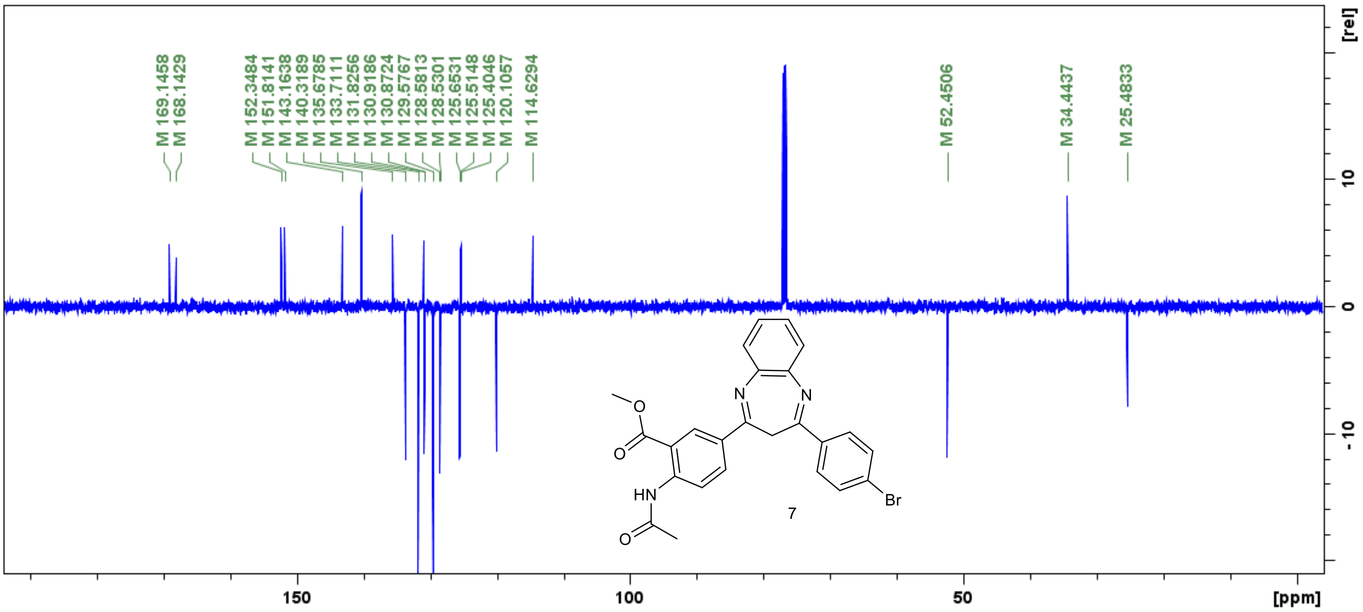

Figure S 3.1. ^{1}H NMR spectrum for compound **8** ($CDCl_3$, 500 MHz)

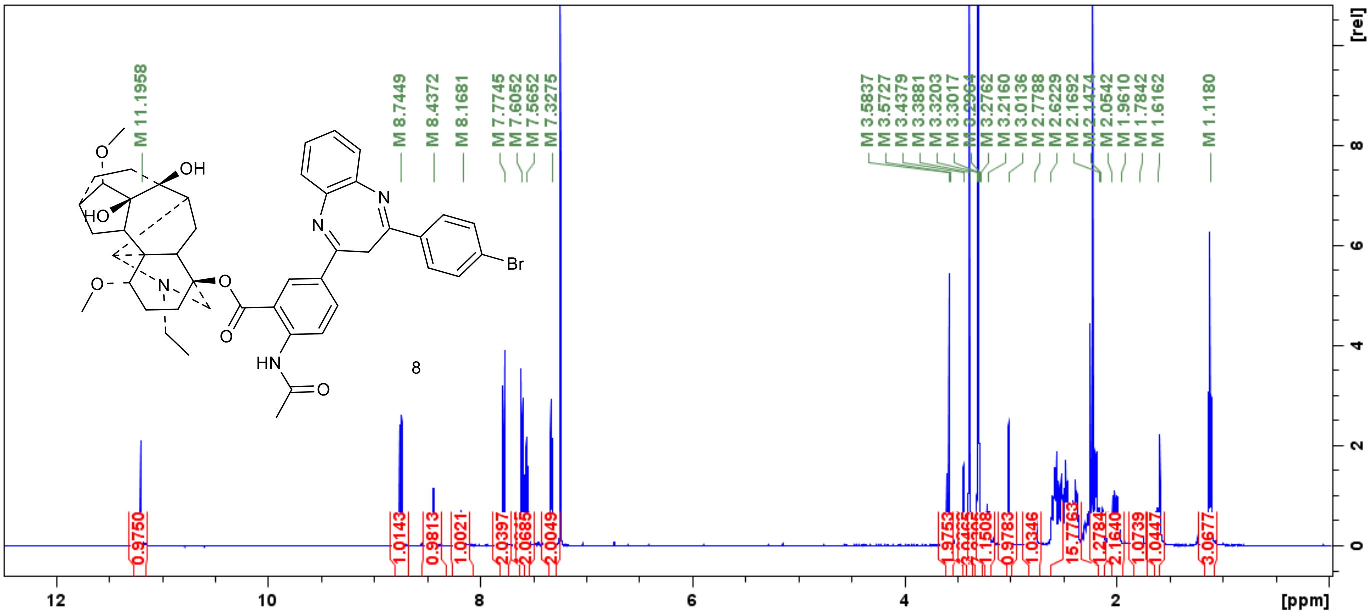

S 3.2.Temperature dependent ^{1}H NMR spectrum for compound **8** recorded in $(CD_3)_2SO$, 400 MHz)

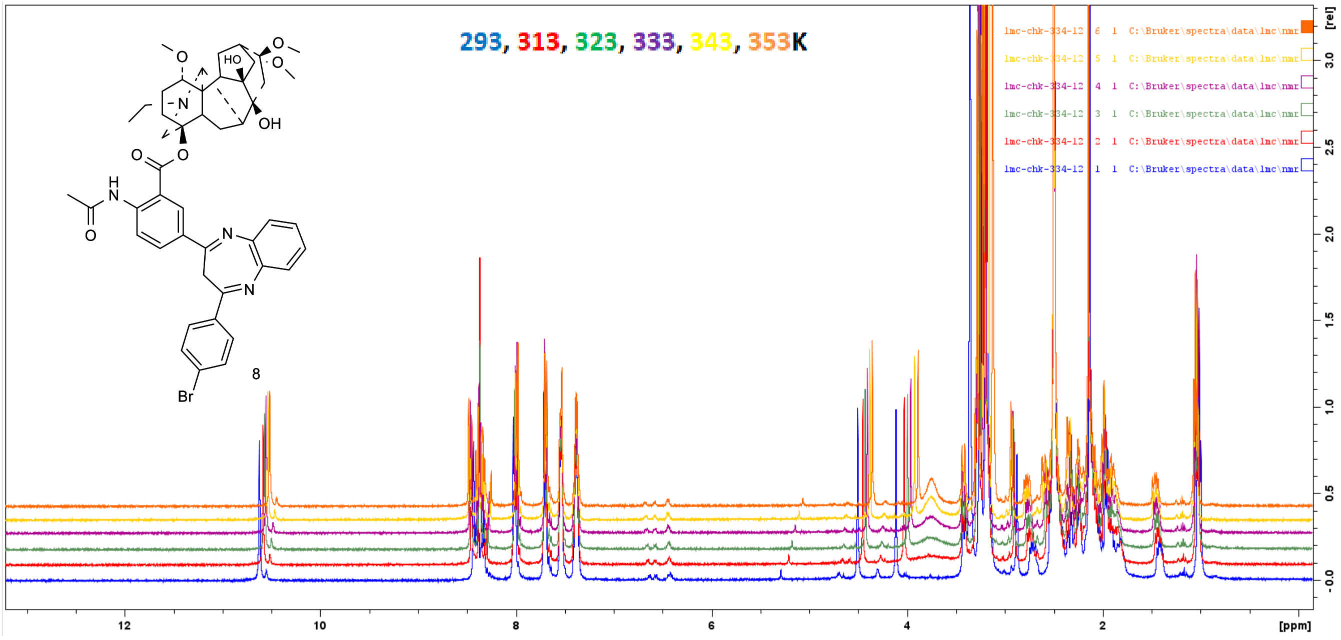

Figure S 3.3. Selected signals from spectra S3.2 for compound **8**.

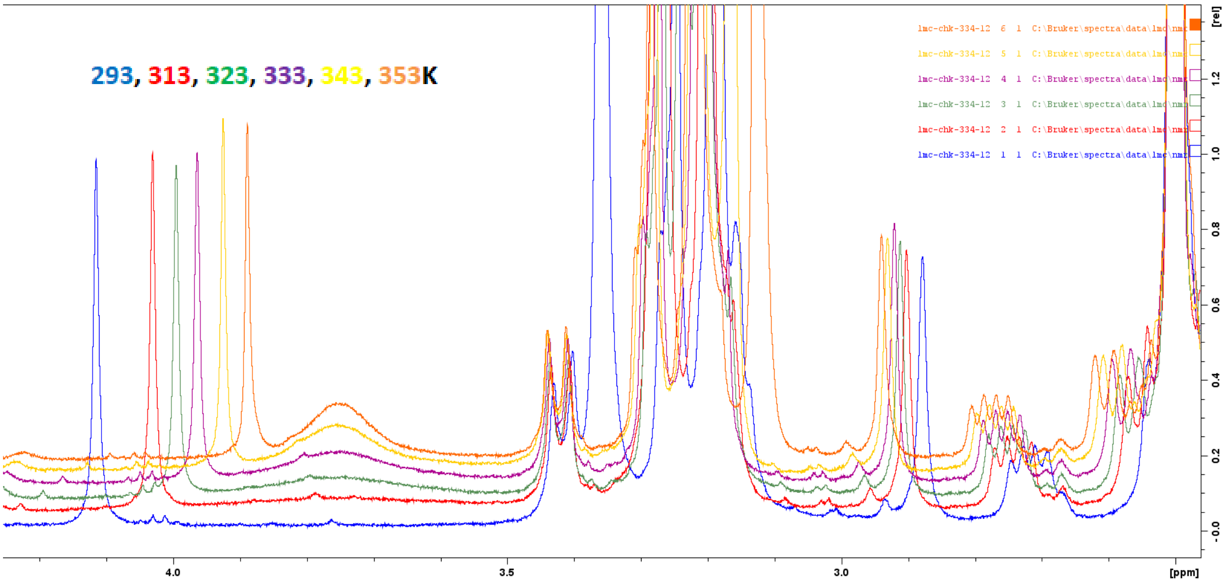

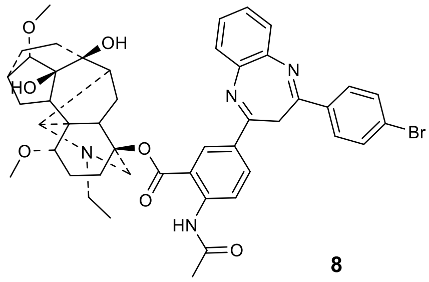

8

Figure S 3.4. **^{13}C NMR** spectrum for compound **8** ($CDCl_3$, 125 MHz)

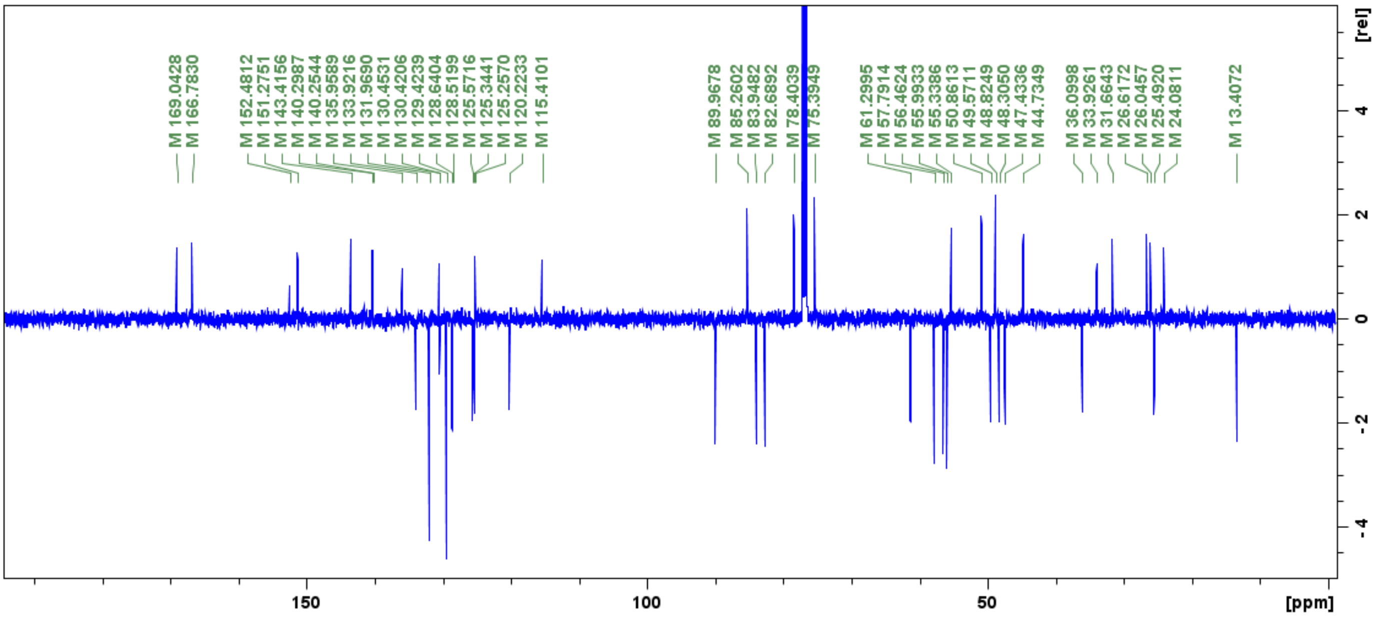

Figure S 4.1. ^{1}H NMR spectrum for compound **9** ($CDCl_3$, 500 MHz)

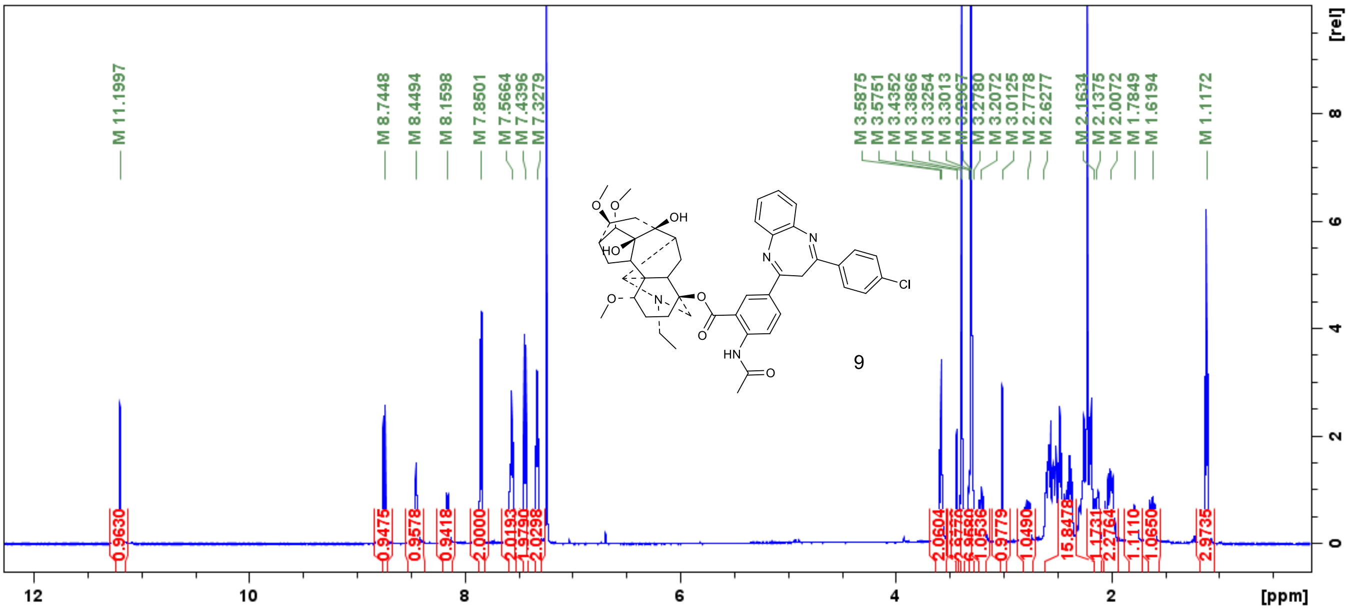

Figure S 4.2. ^{13}C NMR spectrum for compound **9** ($CDCl_3$, 125 MHz)

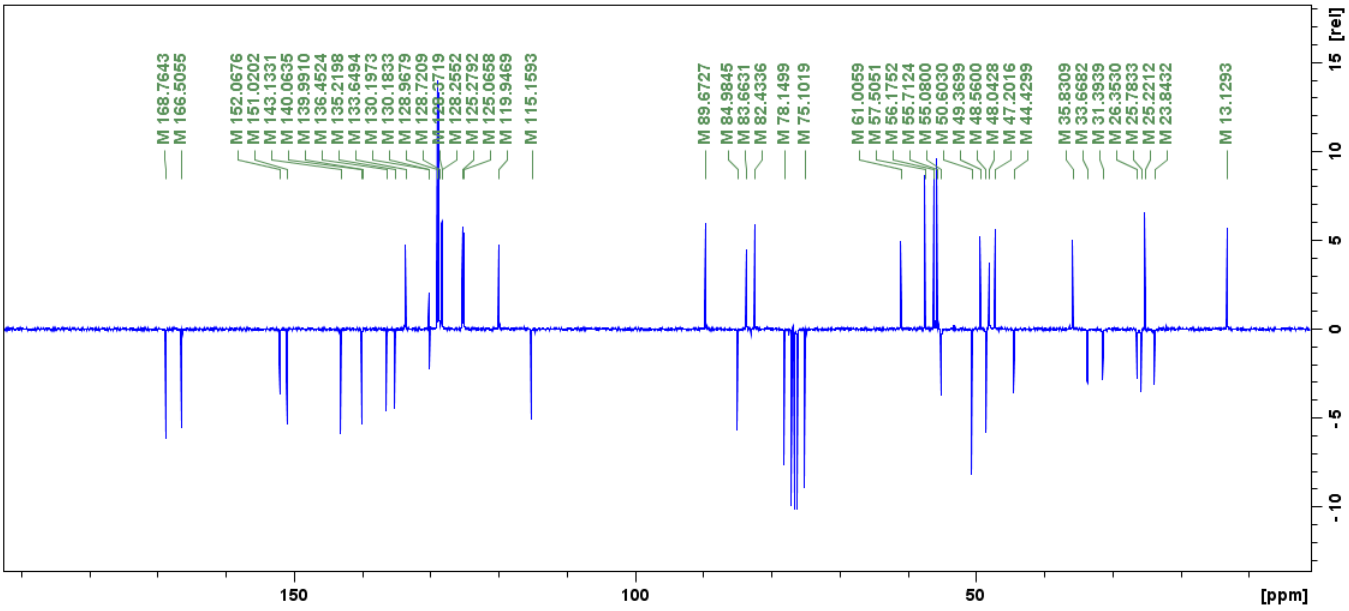

Figure S 5.1. [1]**H NMR** spectrum for compound **10** ($CDCl_3$, 500 MHz)

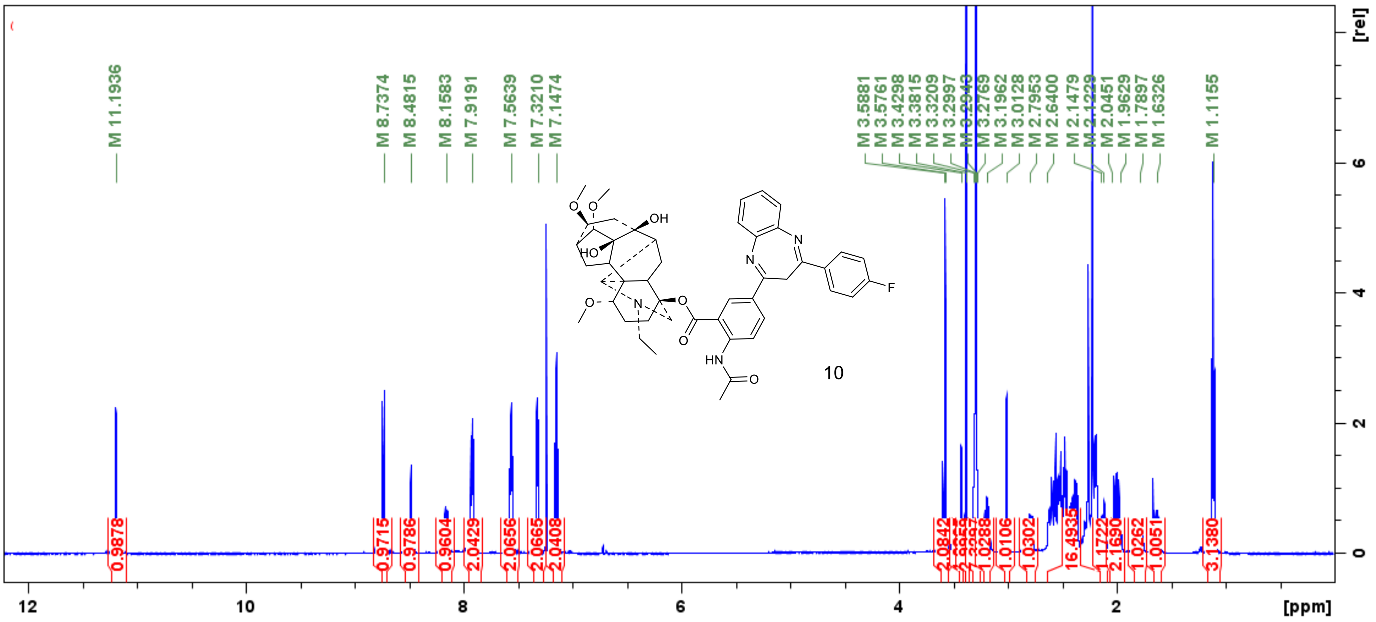

Figure S 5.2. [13]**C NMR** spectrum for compound **10** ($CDCl_3$, 125 MHz)

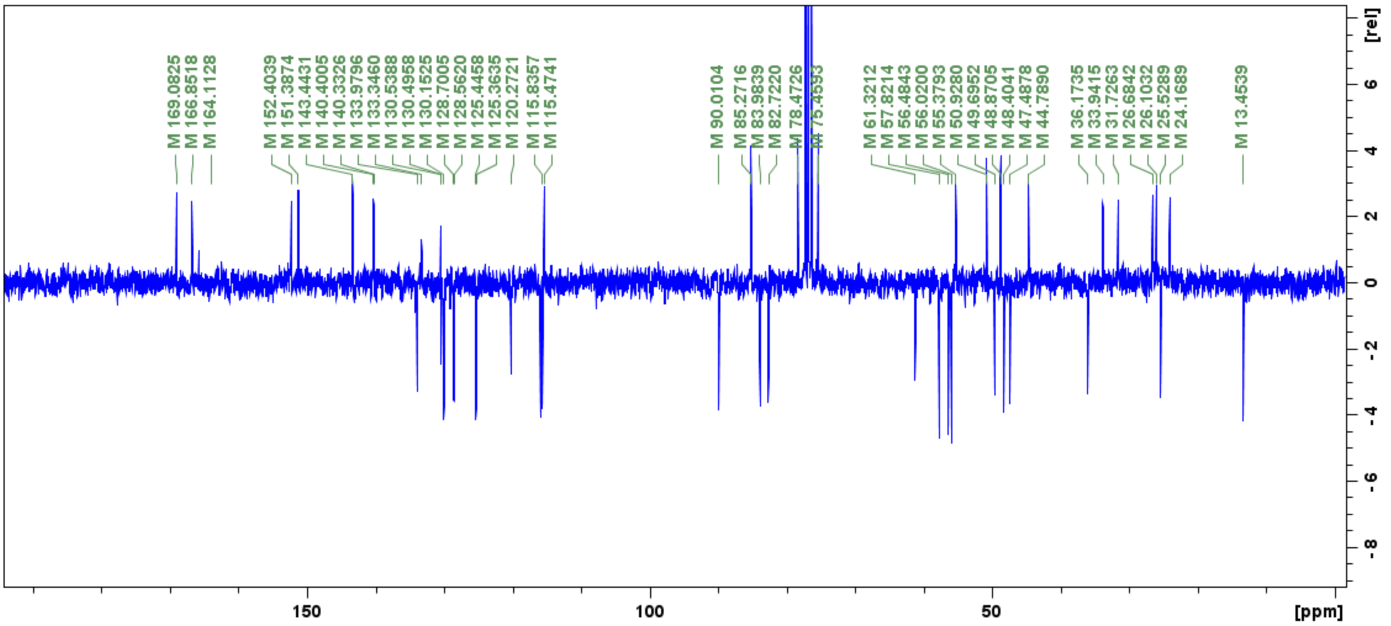

Figure S 6.1. ^{1}**H NMR** spectrum for compound **11** ($CDCl_3$, 400 MHz)

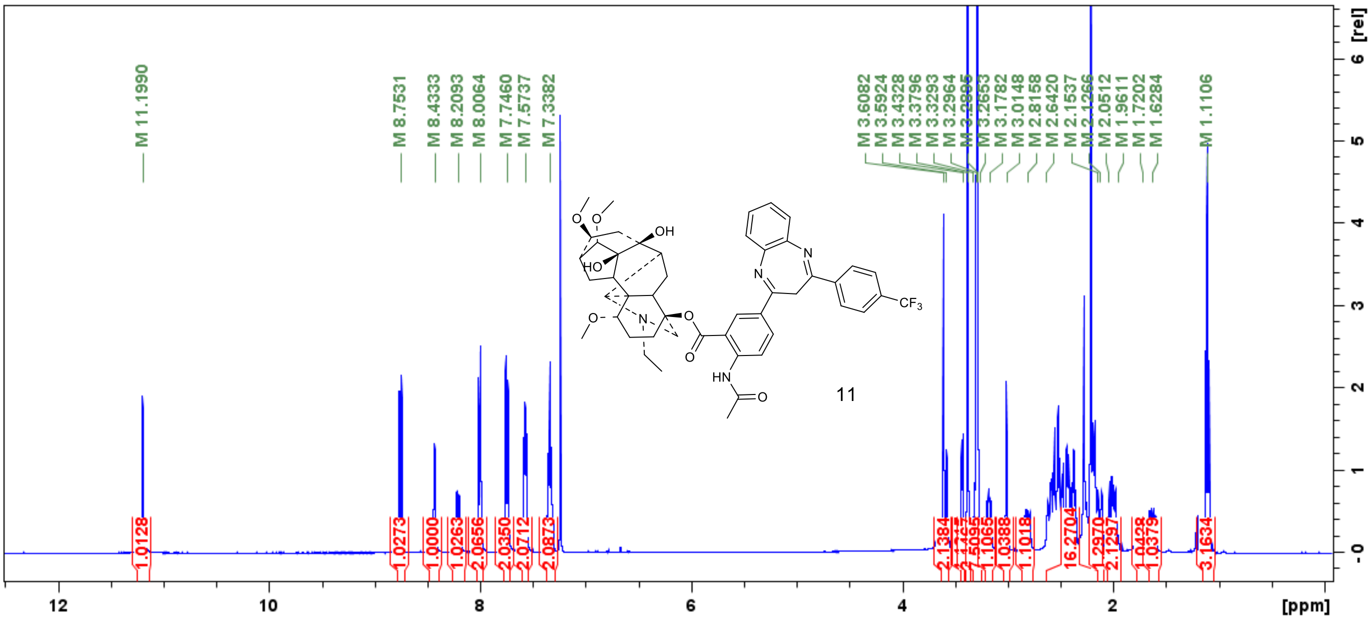

Figure S 6.2. ^{13}**C NMR** spectrum for compound **11** ($CDCl_3$, 100 MHz)

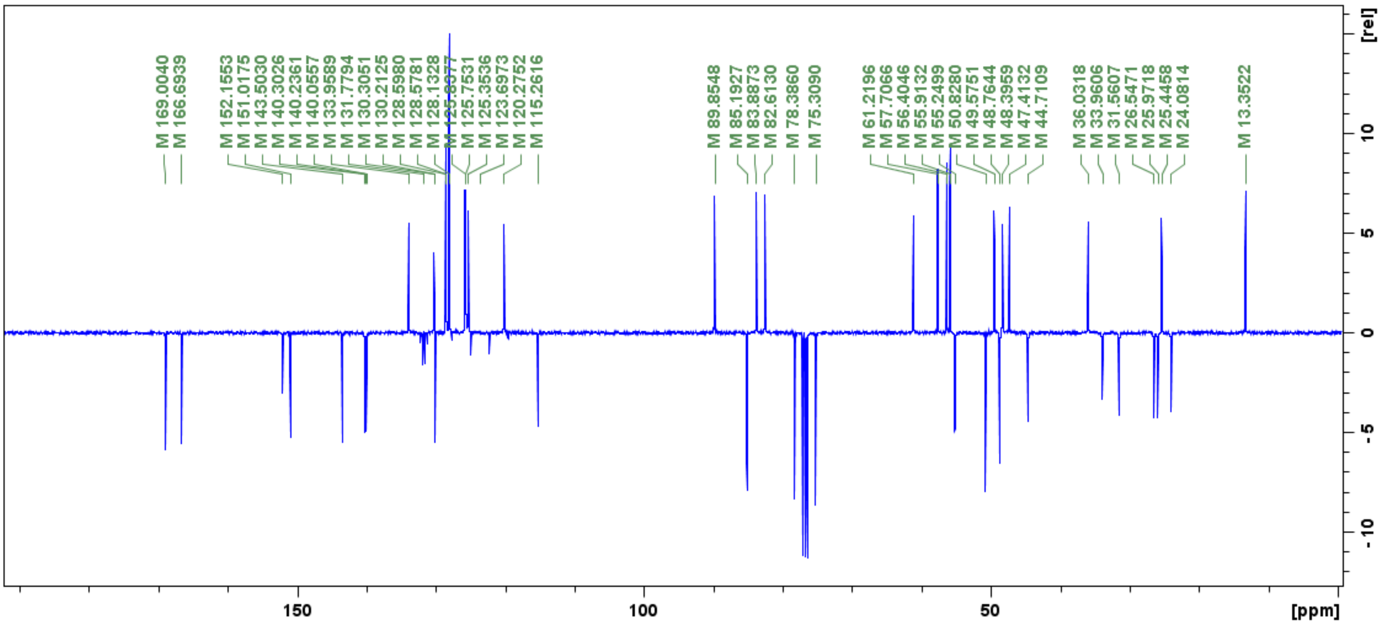

Figure S 7.1. ^{1}H NMR spectrum for compound 12 ($CDCl_3$, 400 MHz)

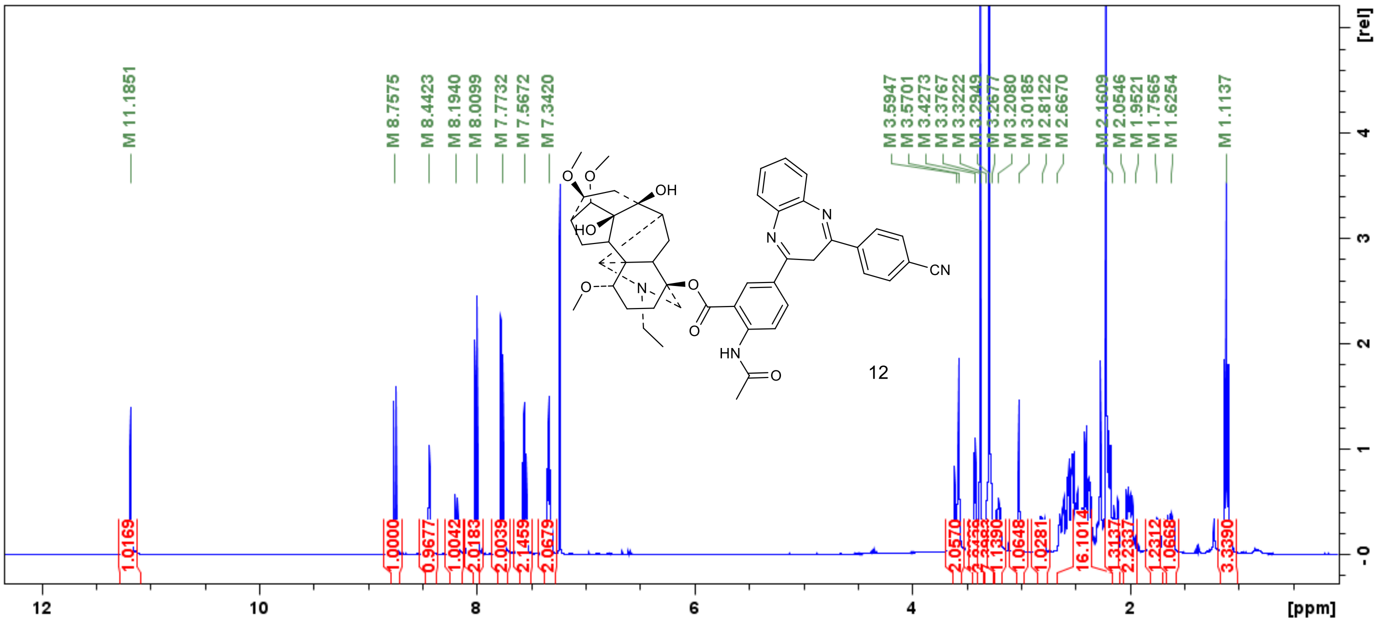

Figure S 7.2. ^{13}C NMR spectrum for compound 12 ($CDCl_3$, 125 MHz)

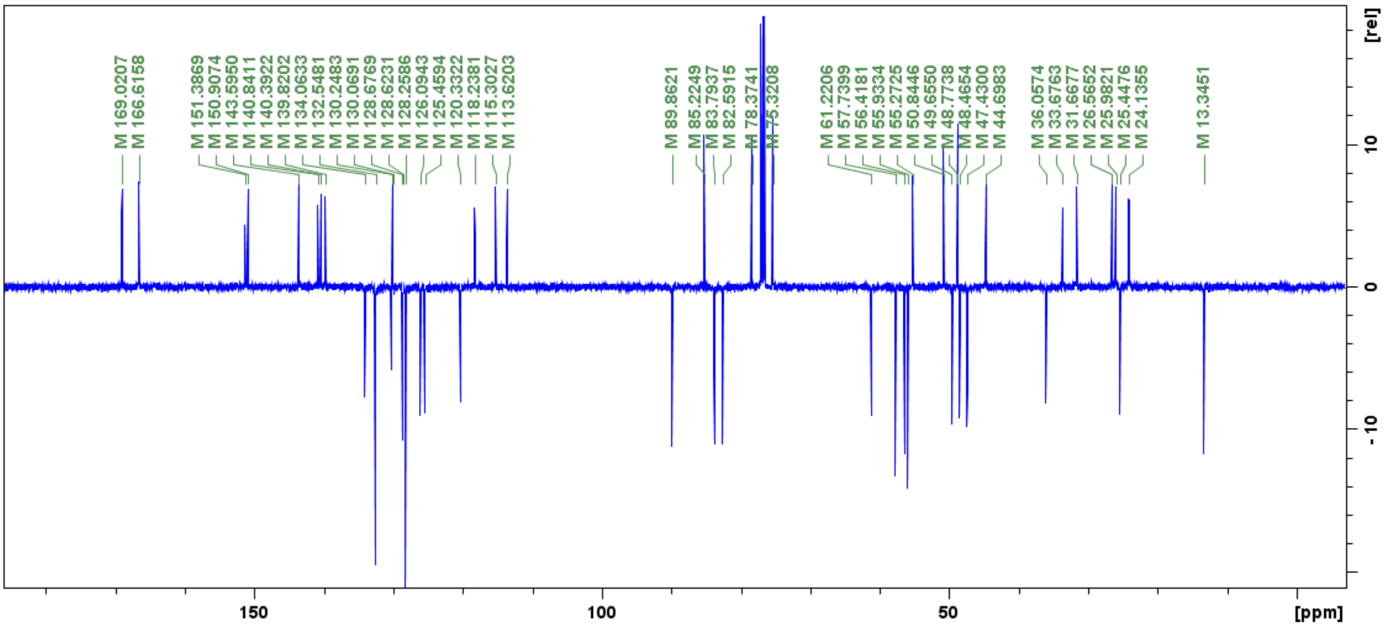

Figure S 8. Effect of compound 8 injection (dose 5mg/kg) on ECG parameters

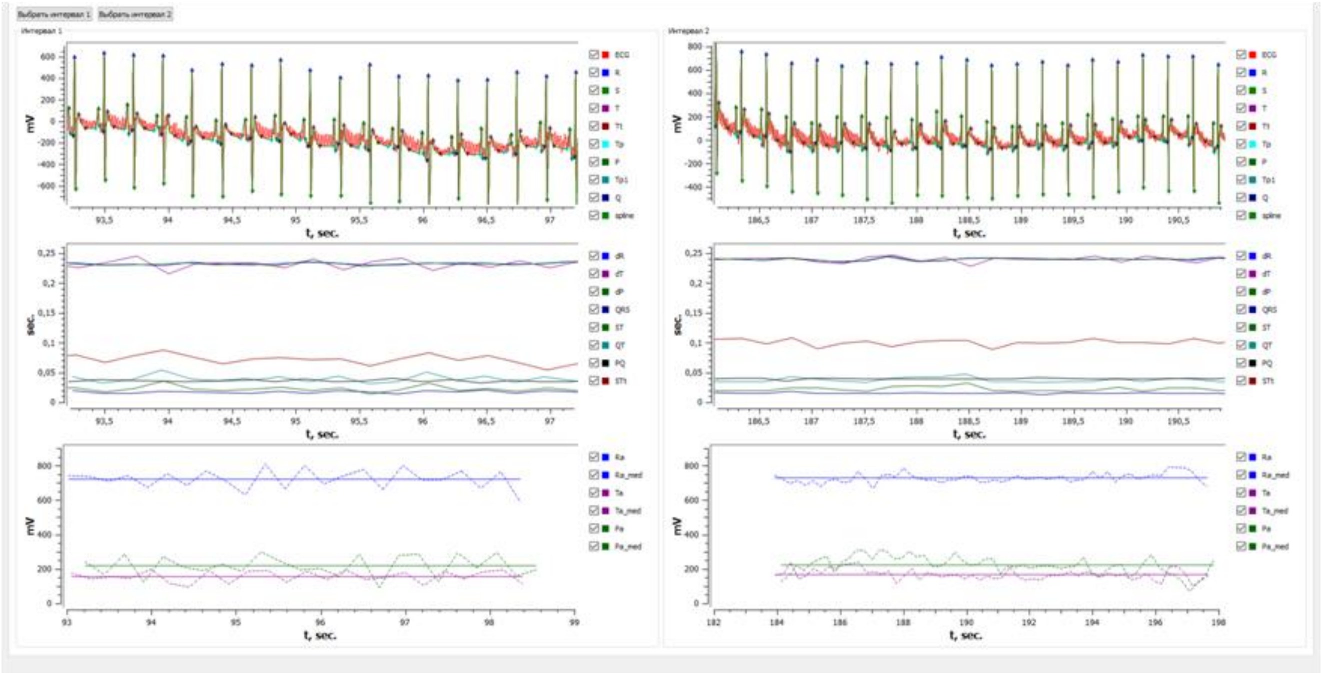

Figure S 9. Effect of compound 10 injection (dose 5mg/kg) on ECG parameters

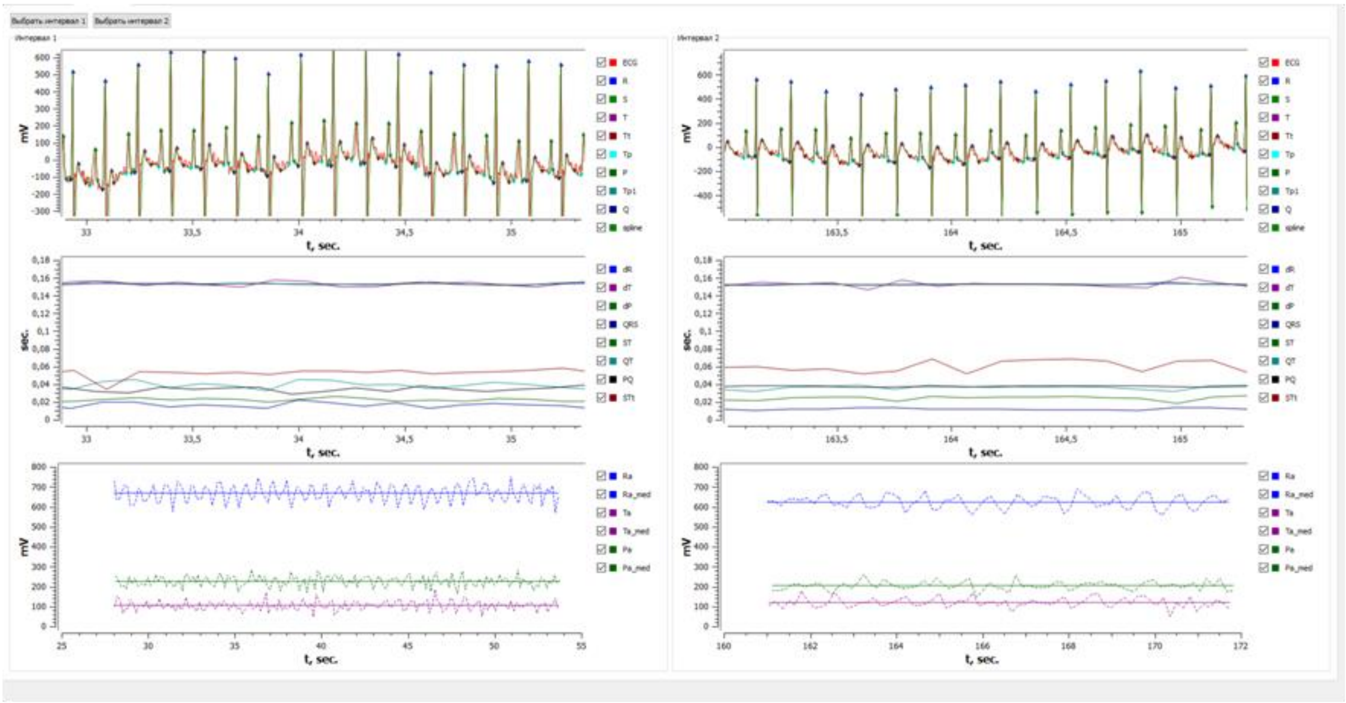

Figure S10. Changes in the rat ECG during administration of compound **8** (10 mg/kg) on a model of calcium chloride arrhythmia.

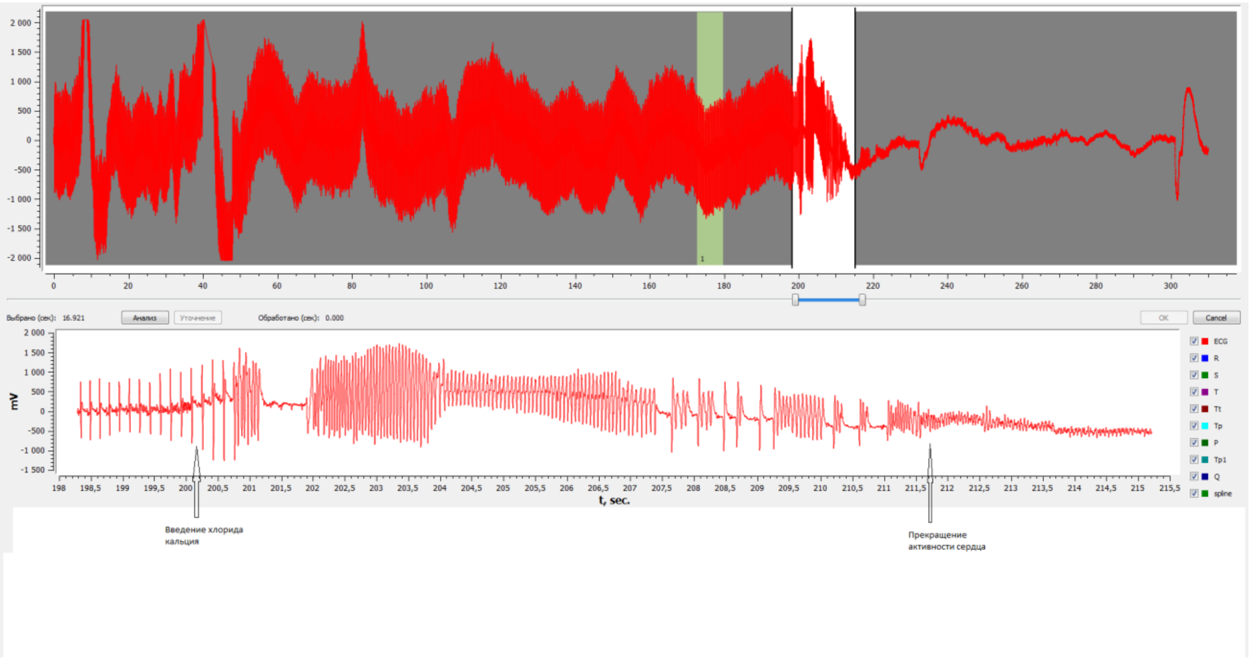

Figure S11. Arrhythmia in rats caused by the introduction of epinephrine and restoration of the rhythm against the background of the administered compound **8** (5 mg/kg). A) native ECG, B) tachycardia after epinephrine administration, C) ECG recovery.

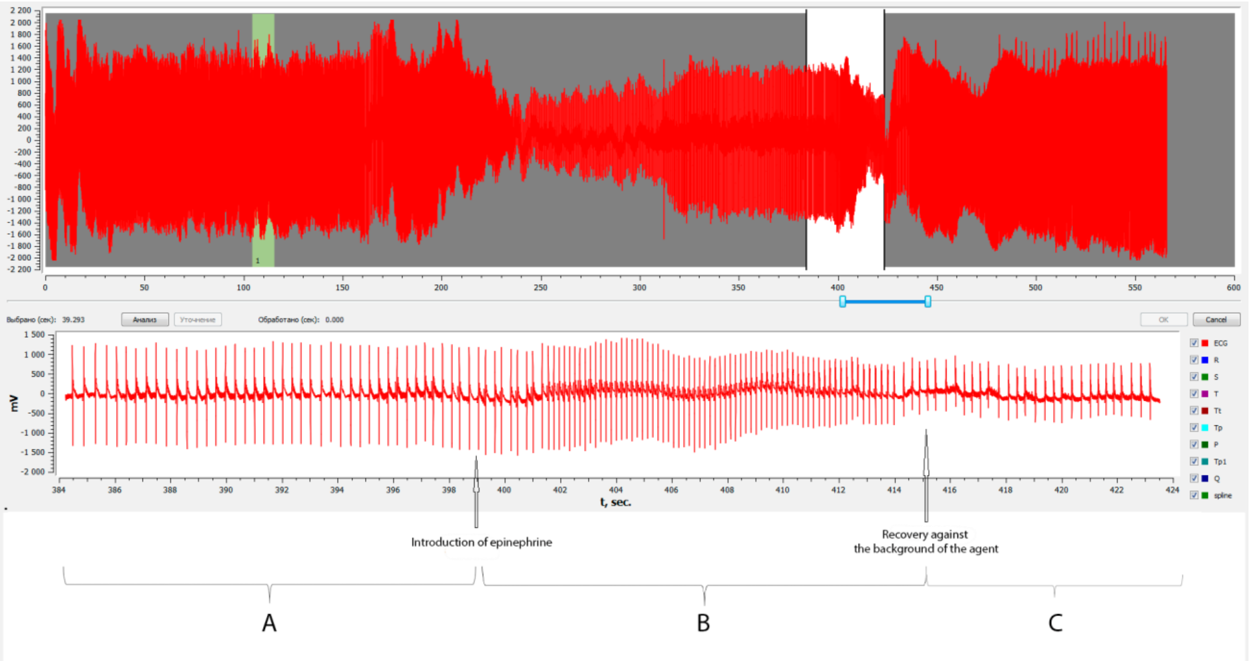

Figure S12. Rat atrium bead alteration after administration of: (1) Epinephrine (2) barium chloride or (3) calcium chloride at the concentration of 10^{-3} M. The peak - injection of the arrhythmogen.

(1)

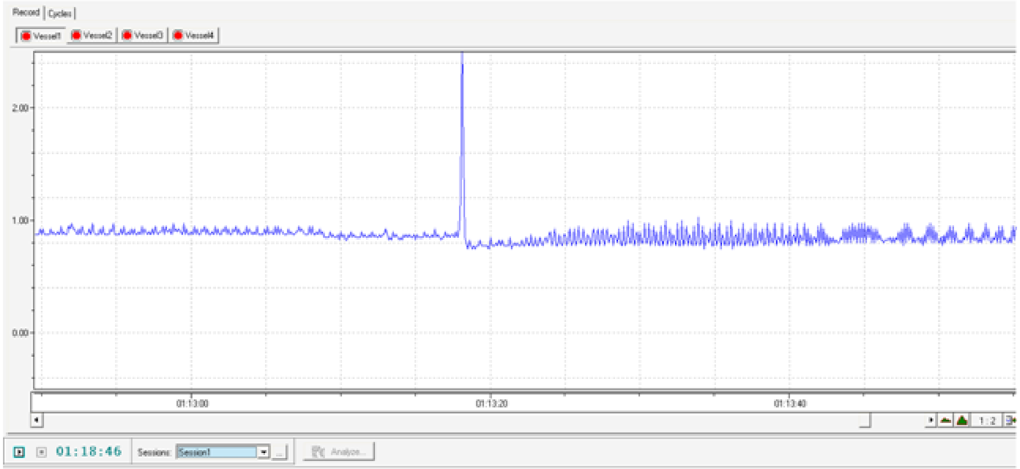

(2)

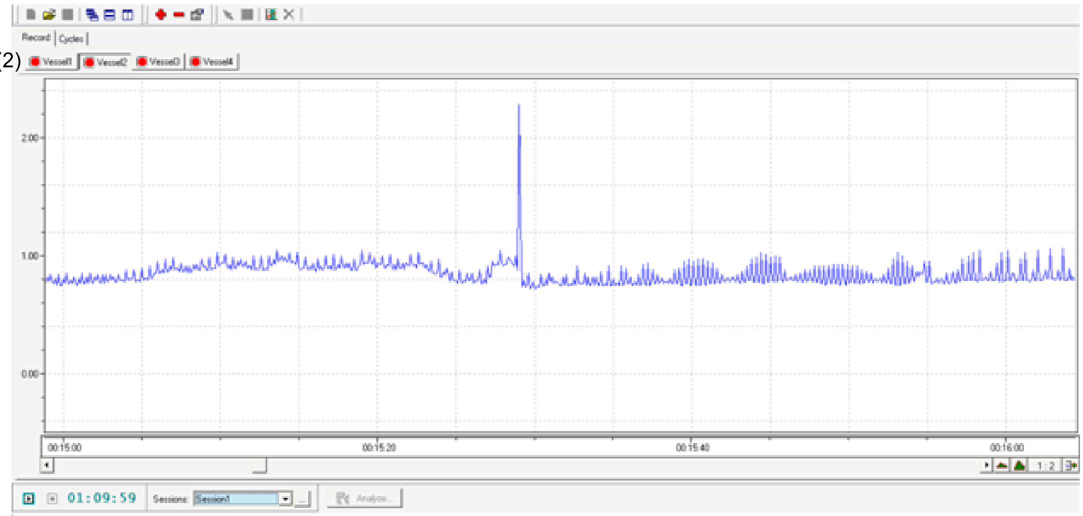

(3)

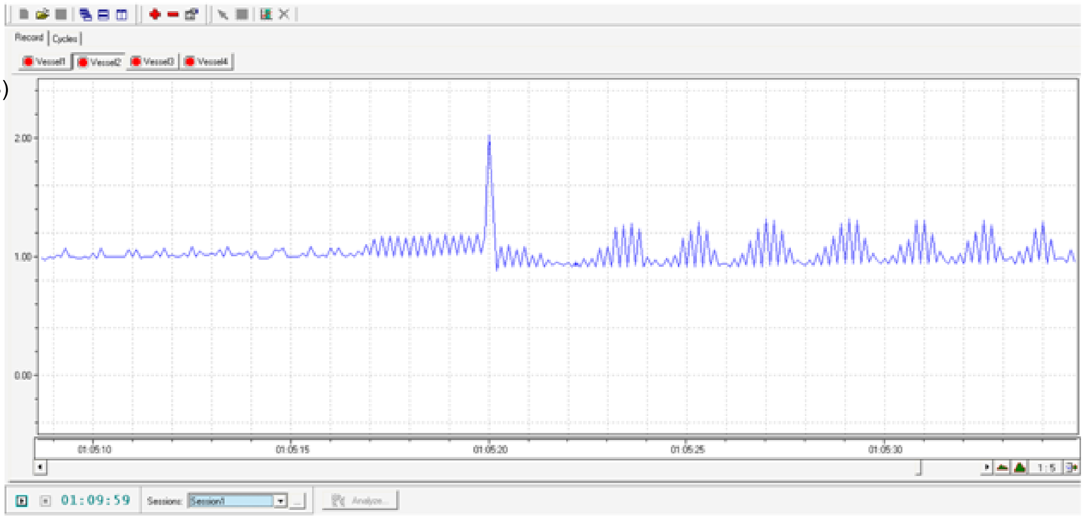

Figure S13. Rat atrium bead alteration after administration of: (1) epinephrine (2) barium chloride or
(3) calcium chloride (concentration of 10^{-3} M) against the background of compounds **8** or **10**
(the peak - injection of the arrhythmogen.

Compound **8**	Compound **10**
(1) Injection of epinephrine after **8** 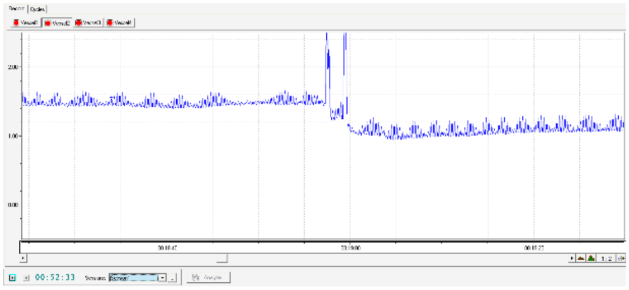	(1) Injection of epinephrine after **10** 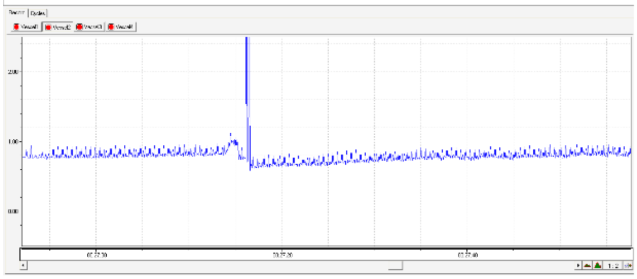
(2) Injection of $BaCl_2$ after **8** 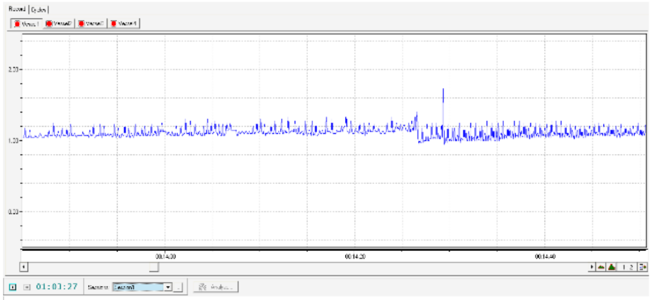	(2) Injection of $BaCl_2$ after **10** 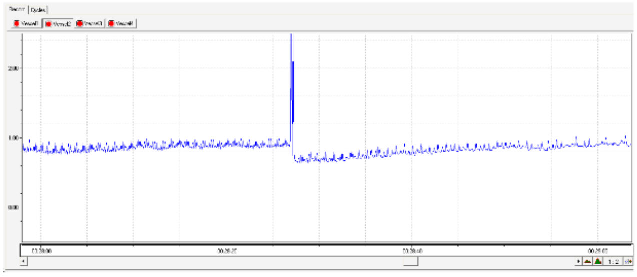
(3) Injection of $CaCl_2$ after **8** 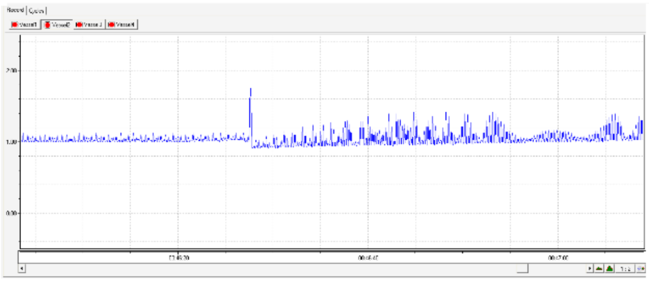	(3) Injection of $BaCl_2$ after **10** 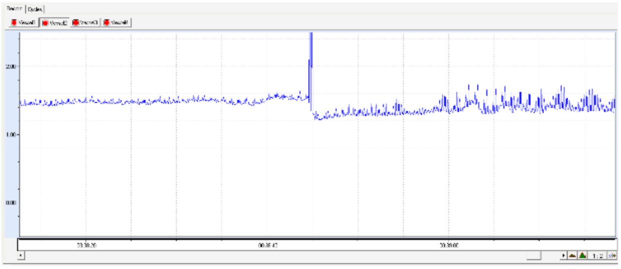

Table S1. Influence of compounds **8** and **10** on ECG parameters in rats in dose 5mg/kg (± shows the spread of the standard error from the mean data).

ECG parameters	dR	dT	dP	QRS	ST	QT	Ra	Ta	Pa
Starting ECG	0.152± 0.005	0.152± 0.007	0.,152± 0.013	0.016± 0.001	0.022± 0.002	0.038± 0.001	667.39± 15.08	104.59± 5.78	226.01± 11.64
With **8**	0.153± 0.002	0.153± 0.009	0.153± 0.008	0012± 0.001	0.023± 0.001	0.035± 0.007	624.49± 20.8	119.53± 6.3	204.91± 6.5
Starting ECG	0.232± 0.0008	0.231± 0.052	0.232± 0.012	0.017± 0.0008	0.022± 0.001	0.039± 0.001	721.04± 30.2	156.62± 9.8	218.12± 15.15
With **10**	0.24± 0.0012	0.24± 0.03	0.24± 0.009	0.015± 0.001	0.022± 0.001	0.037± 0.0001	726.82± 28.8	168.37± 15.128	221.86± 12.8

Table S2. Statistical differences in the amplitude and frequency of contraction of the rat right atrium against the background of compounds **8**,**10** and arrhythmogenes.

Compound	Concen-tration, M	Amplitude, mV		Frequency, Hz	
		Before	After	Before	After
**8**	$10^{-3.}$	0.156*±0.018	0.158±0.004	0.22±0.04	0.20±0001
**10**	$10^{-3.}$	0.18±0.015	0.20±0.009*	0.33±0.05	0.29±0.1
Epinephrine	10^{-3}	0.14±0.023**	0.24±0.045	2.2	3.4
Barium chloride	10^{-3}	0.10±0.06**	0.22±0.01	2.6	3.4
Calcium chloride	$10^{-3.}$	0.18±0.01**	0.34±0.01**	2.6	3.2

*P < 0.05. **P<0.01 before vs. after

Table S3. Statistical differences in the amplitude and frequency of contraction of the rat right atrium after the introduction of arrhythmogen against the background of compounds **8**, **10**.

Com-pound	Epinephrine 10^{-3}M				Barium chloride 10^{-3}M				Calcium chloride 10^{-3}M			
	Amplitude, mV		Frequency, Hz		Amplitude, mV		Frequency, Hz		Amplitude, mV		Frequency, Hz	
	Before	After	Before	After	Before	After	Before	After	Before	After	Before	After
**8**	0.17±0.01	0.18±0.02	2.6	2.8	0.095±0.02*	0.128± 0.01	2.6	1.6	0.095±0.02*	0.24±0.03	2.6	3.2
**10**	0.18±0.07	0.17±0.03	2.2	3.4	0.2±0.018	0.22± 0.04	2.2	3.4	0.38±0.05*	0.98± 0.01	2.2	3.2

*P < 0.05

Figure S14. Diagrams of interactions of flecainide **16** (A - in the electron microscopy model, B - as a result of IFD) and quinidine **17** (C - in the electron microscopy model, D - as a result of IFD) with amino acids of the sodium channel pore. Non-covalent interactions are shown with lines: hydrogen bonds are purple, electrostatic interactions are red, and stacking interactions are green.

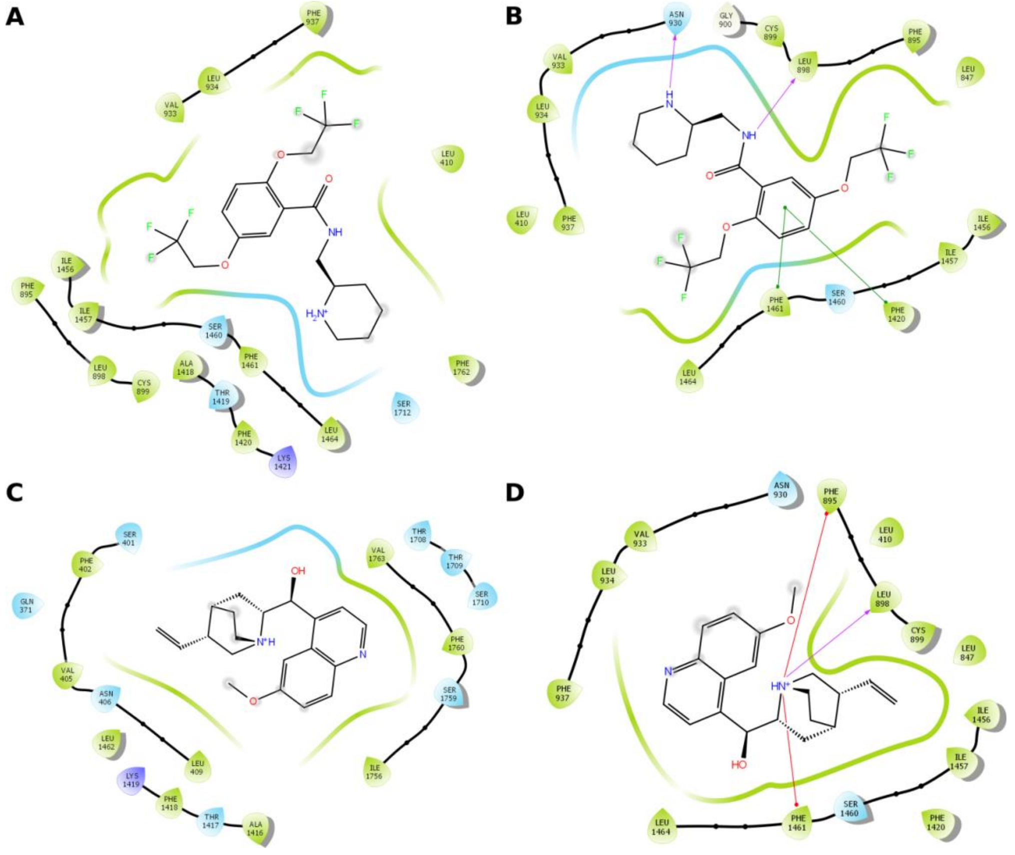

Figure S15. Superposition of sodium channel blockers: green – flecainide **16**, yellow – compound **8**, orange – compound **10**.

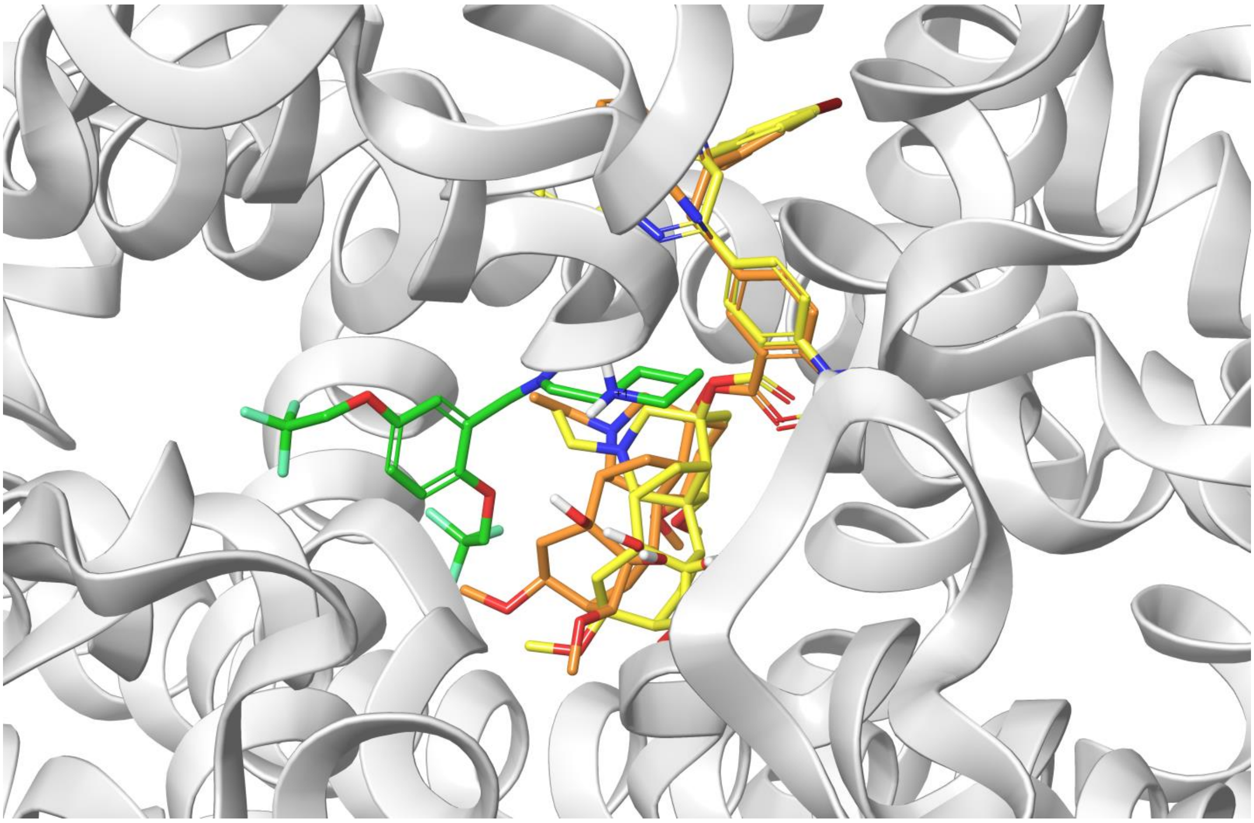

Figure S16. HPLC-UV chromatogram for **4β-{2'-Acetylamino-5'-(4''-(4-bromophenyl)-3H-1,5-benzodiazepine-2''-yl)benzoate}-1α,14α,16β-trimethoxy-20-ethylaconitane-8,9-diol** (**8**)

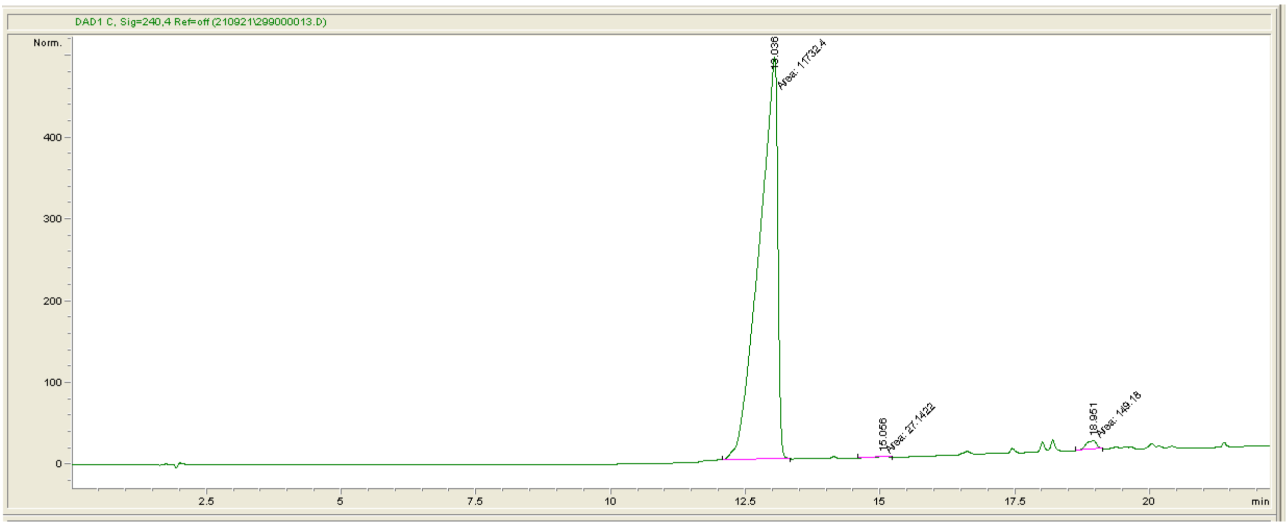

Purity – 98.52%

Retention time - 13,06 мин.

Absorption maximum at wavelength 240 nm.
